# Engineering Allogeneic FE002-Cart Chondroprogenitor Spheroids for Large Knee Chondral Defects: Investigating Microenvironmental Cues for Functional Control, GMP Formulation, and Logistical Viability

**DOI:** 10.3390/pharmaceutics18081032

**Published:** 2026-08-20

**Authors:** Lee Ann Applegate, Farid Hadjab, Sandra Jaccoud, Alexandre Porcello, Virginie Philippe, Nathalie Hirt-Burri, Corinne Scaletta, Brigitte M. Jolles, Dominique P. Pioletti, Robin Martin, Alexis E. Laurent

**Affiliations:** 1 Regenerative Therapy Unit, Lausanne University Hospital, University of Lausanne, CH-1066 Epalinges, Switzerland; lee.laurent-applegate@unil.ch (L.A.A.); sandra.jaccoud@chuv.ch (S.J.); virginie.philippe@chuv.ch (V.P.); nathalie.hirt-burri@unil.ch (N.H.-B.); corinne.scaletta@chuv.ch (C.S.); 2 Center for Applied Biotechnology and Molecular Medicine, University of Zurich, CH-8057 Zurich, Switzerland; 3 Oxford OSCAR Suzhou Center, Oxford University, Suzhou 215123, China; 4 Development Department, Albomed GmbH, D-90592 Schwarzenbruck, Germany; f.hadjab@albomed.eu; 5 Laboratory of Biomechanical Orthopedics, Federal Polytechnic School of Lausanne, CH-1015 Lausanne, Switzerland; 6 Development Department, LOUNA REGENERATIVE SA, CH-1228 Plan-les-Ouates, Switzerland; a.porcello@louna-aesthetics.com; 7 Orthopedic Surgery Service, Fribourg Hospital, CH-1752 Villars-sur-Glâne, Switzerland; robin.martin@h-fr.ch; 8 Department of Musculoskeletal Medicine, Swiss BioMotion Lab, Lausanne University Hospital and University of Lausanne (CHUV-UNIL), CH-1011 Lausanne, Switzerland; brigitte.jolles-haeberli@chuv.ch; 9 Institute of Electrical and Micro Engineering, Ecole Polytechnique Fédérale de Lausanne (EPFL), CH-1015 Lausanne, Switzerland; 10 Manufacturing Department, LAM Biotechnologies SA, CH-1066 Epalinges, Switzerland; 11 Manufacturing Department, TEC-PHARMA SA, CH-1038 Bercher, Switzerland

**Keywords:** advanced therapy medicinal products (ATMP), allogeneic cytotherapies, cartilage tissue engineering, cell formulation, chondrogenesis, good manufacturing practice (GMP), hypoxia, manufacturing process, off-the-shelf implants, three-dimensional spheroids

## Abstract

**Background:** The clinical translation of cell-based therapies for knee articular cartilage repair is fundamentally restricted by the severe biological unpredictability of autologous cell sources, inherent manufacturing bottlenecks, and the rapid phenotypic dedifferentiation of cells expanded in conventional 2D monolayers. To overcome these translational hurdles, this study engineered a scaffold-free, 3D formulation of highly characterized allogeneic FE002-Cart chondroprogenitor spheroids. **Methods:** We systematically investigated the specific microenvironmental cues and Good Manufacturing Practice (GMP) formulation parameters required to direct functional chondrogenesis. The structural and biochemical performance of this allogeneic formulation was benchmarked against multiple primary adult autologous chondrocyte types. Finally, we evaluated the phenotypic resilience of the microtissues in simulated osteoarthritic (OA) environments and investigated both short-term liquid storage and advanced terminal preservation strategies to establish off-the-shelf logistical viability. **Results:** Precise microenvironmental regulation proved to be a critical biological prerequisite. The synergistic combination of physiological hypoxia (2% O_2_) and stringent glucocorticoid limitation (10 nM dexamethasone) induced robust glycosaminoglycan (GAG) deposition and a > 200-fold upregulation of *ACAN* and *COL2*, while suppressing the terminal hypertrophic drift observed in adult chondrocytes. Benchmarking revealed that the allogeneic FE002-Cart formulation substantially mitigates the profound morphological and biochemical unpredictability inherent to adult autologous cell sources. Furthermore, the scaffold-free spheroid geometry yielded a 10-fold increase in GAG production per cell compared to traditional matrix-seeded (MACI) platforms. Transitioning to a GMP-compatible manufacturing process revealed extreme cellular sensitivities; excipients within standard pharmaceutical-grade dexamethasone severely aborted chondrogenic differentiation, emphasizing the necessity of rigorous raw-material qualification. Functionally, the 3D architecture acted as a protective physical shield, sustaining high cellular viability when subjected to severe inflammatory stress and 100% OA patient synovial fluid. Logistically, the viable spheroids maintained matrix integrity and inter-spheroid fusion potential for up to 7 days at ambient temperature in transport medium. Finally, advanced spheroid preservation via lyophilization and high-dose gamma irradiation eliminated biological viability but successfully transitioned the microtissues into highly organized, terminally irradiated matrices capable of heterologous in vitro structural merging. **Conclusions:** These findings define the critical biological thresholds for manufacturing, demonstrate the enhanced in vitro biosynthetic efficiency of 3D allogeneic microtissues compared to specific autologous and matrix-dependent baselines, and establish a highly practical, off-the-shelf logistical framework for the regenerative treatment of large knee chondral defects.

## 1. Introduction

The limited intrinsic repair capacity of articular cartilage presents a considerable clinical challenge, as unaddressed defects progressively result in the development of osteoarthritis (OA) [1,2]. The latter is a debilitating degenerative joint disease that currently affects hundreds of millions of individuals worldwide and imposes a massive socioeconomic burden through direct healthcare utilization and severe productivity loss [3,4,5,6,7,8,9]. Current cell-based therapeutic strategies, such as autologous chondrocyte implantation (ACI), attempt to address this tissue deficit but are fundamentally hampered by significant donor-site morbidity and the rapid phenotypic dedifferentiation of expanded chondrocytes in conventional 2D in vitro cultures [1,9,10,11,12,13,14,15]. This method typically utilizes biomaterials (e.g., decellularized matrices, collagen, or alginate) to facilitate cell adhesion and bridge physical defect gaps [16]. Alternatively, modular tissue engineering leverages self-assembling 3D cell spheroids as independent building blocks that can natively fuse to bridge structural defect gaps without exogenous materials [17,18,19,20]. Furthermore, patient-derived autologous cell therapies suffer from profound biological unpredictability, as the regenerative capacity and phenotypic stability of these cells are heavily dictated by donor age, genetic background, and pre-existing joint comorbidities [21,22]. Consequently, achieving standardized, large-scale commercial manufacturing for these autologous implants remains technically challenging and susceptible to supply chain and regulatory hurdles [23,24].

To overcome the severe limitations of autologous cell harvesting and 2D expansion, allogeneic cell sources have emerged as a highly promising translational frontier, offering the distinct advantage of standardized, readily available biological treatments that represent the consensus future of operative cartilage repair [22,25,26,27]. The well-characterized allogeneic human chondroprogenitor cell type, FE002-Cart, has previously demonstrated clinical suitability when utilized with supportive collagen matrices [28]. However, transitioning this cellular platform from a matrix-dependent system to a highly organized, scaffold-free 3D cell spheroid formulation represents a promising Advanced Therapy Medicinal Product (ATMP) delivery strategy to be investigated [10,29,30,31]. The spheroid platform offers distinct theoretical advantages over both existing allogeneic cell suspensions and matrix-assisted systems [28]. This approach aims to eliminate the need for an exogenous matrix, thereby avoiding potential clinical risks associated with suboptimal scaffold degradation or immune responses, while facilitating robust chondrogenic redifferentiation and autonomous extracellular matrix (ECM) synthesis [10,29,30,31]. By utilizing scaffold-free cell aggregation techniques, these spheroids naturally self-assemble into microtissues that much more accurately simulate the native living environment of chondrocytes than isolated cells [32]. This autonomous assembly is actively driven by the high cellularity and 3D spatial arrangement that inherently mimic natural cell–cell and cell–matrix interactions [33,34,35].

Furthermore, compared to unstandardized allogeneic cell pools or highly variable primary autologous sources, banked FE002-Cart cells actively resist phenotypic drift, securing the lot-to-lot biological consistency required for obtaining appropriate risk-benefit ratios [28]. Globally, while this represents a scaffold-free approach that has already demonstrated favorable safety profiles and clinical efficacy in autologous trials [36,37,38,39,40,41], the specific microenvironmental thresholds and GMP formulation parameters required to optimally engineer this allogeneic FE002-Cart platform remain a critical, unaddressed knowledge gap. Importantly, the in vitro functional payload and chondrogenic potential of these 3D cellular formulations, often predicted by the baseline expression of specific matrix proteins like aggrecan, are highly sensitive to the precise microenvironmental conditions applied during the manufacturing process [42]. In their native joint environment, chondrocytes reside in a profoundly hypoxic niche, making ambient oxygen tension a critical, active regulator of cellular metabolism and matrix assembly [34,43]. Therefore, defining the precise physiological oxygen thresholds required to drive optimal ECM synthesis in vitro while preventing unwanted terminal hypertrophy remains a key requirement for standardizing this specific FE002-Cart 3D microtissue manufacturing process [44,45]. Concurrently, the biochemical formulation of the differentiation media, specifically the precise concentration of synthetic glucocorticoids, acts as a master regulator of chondrogenic signaling cascades that necessitates rigorous optimization to avoid off-target differentiation [46,47].

Beyond baseline biological optimization, translating cell-based therapies to the clinic requires a complex transition from research and development protocols to Good Manufacturing Practice (GMP)-compatible formulations. This transition presents a substantial scientific challenge [48]. The necessary substitution of optimized research reagents with pharmaceutical-grade alternatives introduces excipients and subtle formulation variations that can alter intricate cellular signaling pathways and potentially impair chondrogenic differentiation [46,47].

Furthermore, the real-world clinical utility of any living cellular biologic is heavily constrained by its phenotypic stability in vivo and its logistical supply chain. Upon surgical implantation, therapeutic cells are subjected to a harsh, catabolic microenvironment characterized by pro-inflammatory mediators and degradative enzymes within the osteoarthritic joint space [43,49]. Traditional therapies utilizing monodispersed cell suspensions often face challenges in these conditions, exhibiting rapid cell death and insufficient tissue retention [50,51]. Therefore, a robust 3D spheroid architecture is hypothesized to act as a protective formulation; by maintaining a dense, self-assembled ECM network, spheroids may shield therapeutic cells from specific external stimuli, allowing them to maintain matrix integrity even when exposed directly to inflammatory conditions and OA synovial fluid, a resilience that has recently been demonstrated to preserve cell viability and tissue-fusion capacity in other severe degenerative microenvironments [24,52,53,54].

Additionally, for an allogeneic product to truly fulfill its “off-the-shelf” potential, it must demonstrate substantial logistical viability. Establishing simplified, highly specific storage conditions that preserve spheroid viability, structural integrity, and tissue-fusion capacity over extended periods is a paramount translational objective, as standard long-term preservation methods often struggle to maintain high cellular recovery and membrane integrity, which can delay or impair the intrinsic fusion kinetics required for scaffold-free assembly [55,56]. This logistical buffer is fundamentally essential for streamlining clinical workflows and distribution prior to patient implantation. While short-term liquid storage provides a necessary logistical buffer, alternative preservation methods are crucial for maximizing the ultimate off-the-shelf utility of the product.

Relative to prior literature, the novelty of this study spans three specific domains. First, unlike previous matrix-assisted FE002-Cart applications, we define the precise microenvironmental thresholds, specifically physiological hypoxia and stringent glucocorticoid limits, required to drive autonomous ECM synthesis and prevent hypertrophic drift in a scaffold-free 3D state [28]. Second, advancing beyond previous spheroid studies, we quantify the severe biological sensitivities of these microtissues to pharmaceutical-grade excipients during the critical transition to a GMP-compliant manufacturing process [17,19,29]. Finally, diverging from conventional cartilage ATMP cold-chain logistics, we establish ambient-temperature storage limits and explore the feasibility of lyophilization and terminal sterilization for potential off-the-shelf utility [1,7,9].

Addressing these specific manufacturing and translational knowledge gaps, this study aimed to develop and characterize a scaffold-free formulation of allogeneic FE002-Cart fetal chondroprogenitor spheroids specifically targeted for the regenerative treatment of large knee chondral defects. First, we sought to determine the precise microenvironmental cues required for optimal functional control, specifically investigating the profound biological impact of varying hypoxic levels and dexamethasone concentrations. Second, we systematically investigated the complex transition from a research-grade to a GMP-compliant manufacturing process, thoroughly analyzing raw material sensitivities.

Third, to contextualize the functional performance of this allogeneic platform, we systematically benchmarked its morphological and biochemical consistency against adult autologous chondrocytes. Finally, to establish true clinical viability, we characterized the phenotypic resilience of these engineered spheroids in challenging osteoarthritic environments, identified optimal short-term liquid storage/transport parameters, and explored advanced terminal preservation via lyophilization and gamma irradiation to assess their potential as sterile, integrative scaffolds.

## 2. Materials and Methods

### 2.1. General Reagents and Consumables

Unless otherwise specified, all reagents utilized were of analytical or cell culture grade. The general materials used throughout the study are categorized as follows:

Cell culture and differentiation reagents: Dulbecco’s Modified Eagle Medium (DMEM), Opti-MEM, L-glutamine, TrypLE™, penicillin-streptomycin, and dexamethasone were procured from Thermo Fisher Scientific (Waltham, MA, USA). Fetal bovine serum (FBS) and vitamin C 2-phosphate (VitCp) were provided by Merck (Darmstadt, Germany). Additional penicillin-streptomycin was sourced from Biowest (Nuaillé, France), and ITS 100× supplements from PAN-Biotech (Aidenbach, Germany). Recombinant human TGF-β1 and TGF-β3 were obtained from PeproTech (London, UK). Recombinant TNF-α, IL-1β, and IL-6 were obtained from R&D Systems (Minneapolis, MN, USA).

Buffers and general chemicals: Purified water, phosphate-buffered saline (PBS), and 0.9% NaCl solutions were supplied by Bichsel (Unterseen, Switzerland). Ultrapure water, isopropanol, and sodium chloride were obtained from Thermo Fisher Scientific (Waltham, MA, USA). Dibasic and monobasic sodium phosphate, alongside papain, were purchased from Sigma-Aldrich (Buchs, Switzerland). Sodium acetate, EDTA, cysteine HCl, and low-melting-point agarose were sourced from Merck (Darmstadt, Germany). Formulation stabilizers, specifically saccharose and dextran 40,000, were provided by PanReac AppliChem (Darmstadt, Germany) and Pharmacosmos (Wiesbaden, Germany), respectively.

Biochemical assays and protein analysis: BCA protein assay kits, MTT reagent, β-mercaptoethanol, PMSF, NuPAGE Bis-Tris 4–12% gels, MOPS buffer, loading buffer, DTT, antioxidant, PageRuler protein ladders, formaldehyde, glutaraldehyde, potassium ferricyanide, and potassium ferrocyanide were all supplied by Thermo Fisher Scientific (Waltham, MA, USA). Trypan Blue was obtained from Merck (Darmstadt, Germany), and X-gal powder from Chemie Brunschwig (Basel, Switzerland). Glycosaminoglycan (GAG) quantification was performed using Blyscan-sulfated GAG assay kits (BioColor, Carrickfergus, UK).

Molecular biology reagents: KAPA SYBR Fast qPCR master mix was acquired from Roche (Basel, Switzerland). Telomerase activity quantification qPCR assay kits were sourced from ScienCell (Carlsbad, CA, USA).

General consumables and lyophilization materials: General cell culture vessels, assay tubes, and plastic assay surfaces were obtained from Greiner BioOne (Frickenhausen, Germany) and TPP (Trasadingen, Switzerland). Cell counting was performed using C-Chip Neubauer hemocytometers (NanoEntek, Seoul, Republic of Korea), and media filtration utilized Millipore Stericups (Merck, Darmstadt, Germany). PCR consumables, including MicroAmp fast 96-well reaction plates and optical adhesive covers, along with 96-well PCR plates, were supplied by Thermo Fisher Scientific (Waltham, MA, USA). Materials for the freeze-drying processes included Lyoprotect bags (Teclen, Oberpframmern, Germany), lyophilization vials (Schott, Mainz, Germany), and lyophilization stoppers (Flaver, Reinach, Switzerland).

### 2.2. Instruments and Equipment

Spectrophotometric and colorimetric analyses were conducted using a Varioskan LUX multimode microplate reader (Thermo Fisher Scientific, Waltham, MA, USA). Quantitative real-time polymerase chain reaction (RT-qPCR) amplification and fluorescence detection were carried out on a StepOne Real-Time PCR System (Thermo Fisher Scientific, Waltham, MA, USA). Histological and immunohistochemical image acquisition was performed utilizing an IX81 inverted fluorescence microscope (Olympus, Tokyo, Japan). Electrophoretic protein gel visualization and documentation were achieved with a Uvitec Mini HD9 gel imager (Cleaver Scientific, Rugby, UK). Finally, the controlled lyophilization of the formulated spheroid samples was executed using a LyoBeta Mini freeze-dryer (Telstar, Terrassa, Spain).

### 2.3. Biological Material Sources

The primary biological model for this study utilized the allogeneic FE002-Cart chondroprogenitor cell type, which consists of banked primary human diploid cells derived from a clinical-grade source. These cells were procured and manufactured under the Swiss progenitor cell transplantation program and were maintained as cryopreserved master and working cell banks. For all experimental procedures, FE002-Cart chondroprogenitors were thawed and cultured in a complete growth medium comprising DMEM supplemented with 2 mM L-glutamine and 10% fetal bovine serum (FBS). The cells were expanded in 2D monolayer cultures under either normoxic or defined hypoxic conditions, depending on the specific experimental design, and were consistently utilized for experiments between in vitro passage levels 6 and 7.

To evaluate cell donor variability and provide a comparative baseline for spheroid formation, primary adult human chondrocytes were also investigated. Healthy adult chondrocytes were obtained commercially (Innoprot, Derio, Spain). These cells were expanded in α-MEM supplemented with L-glutamine, 10% FBS, penicillin-streptomycin, 10 mM HEPES, 1× non-essential amino acids (NEAA), 1 ng/mL TGF-β1, and 5 ng/mL FGF-2, and were utilized for experiments between passage levels 4 and 5. Additionally, patient-derived primary articular chondrocytes were procured from the biobank of the DAL at the Centre Hospitalier Universitaire Vaudois (CHUV, Lausanne, Switzerland, BB_029_DAL, 2 February 2022). Demographic data for these adult donors are summarized in Table 1.

All donors were participants in a local clinical trial for cartilage lesions of the knee treated by second-generation ACI (NCT04296487 trial). They presented well-demarcated cartilage defects and no degenerative osteoarthritis. All patients fulfilled the inclusion criteria for the corresponding clinical trial. Patient selection for the present study was not based on additional criteria and selection was based on availability of surplus frozen cell stocks. The cells were derived under GMP from the healthy cartilage biopsies harvested for autologous cell therapy preparation and were provided as cryopreserved aliquots [28]. For the needs of the study, these clinical samples were expanded in DMEM containing 2 mM L-glutamine with 10% FBS and were utilized for experiments between passage levels 3 and 4.

Finally, to accurately simulate the pathological and catabolic microenvironment of the diseased joint for functional resilience assays, human synovial fluid was procured from patients diagnosed with grade IV osteoarthritis, also sourced from the DAL biobank (BB_029_DAL, 2 February 2022).

### 2.4. Effect of Oxygen Levels on 2D Cell Culture

#### 2.4.1. Cell Proliferation Assays

Cryopreserved FE002-Cart cells were thawed and initially expanded in T75 culture flasks utilizing complete growth medium. The cultures were allocated to either standard normoxic conditions or strictly regulated hypoxic environments (2%, 5%, or 10% O_2_ with 5% CO_2_ at 37 °C), with culture media exchanged twice weekly. Upon reaching confluence, the cells were dissociated and subsequently seeded at a defined density of 1500 cells/cm^2^ into 6-well tissue culture plates (Greiner, Frickenhausen, Germany), with all experiments performed in triplicate.

To rigorously evaluate the specific impact of ambient oxygen tension on expansion rates, the seeded plates were continuously maintained within their respective pre-assigned normoxic or hypoxic incubation environments. Cellular proliferation kinetics were quantitatively monitored by sequentially harvesting the cells at designated timepoints (D0, D2, D5, D7, and D8) and determining the absolute cell numbers via hemocytometer counting. The mean total cell count per well was subsequently calculated for each condition, facilitating the generation of comprehensive proliferation curves to assess population expansion over time.

#### 2.4.2. Cellular Active Substance Characterization: Multiplex Proteomic Analyses

To comprehensively characterize the cellular active substance, soluble protein identification and quantification were conducted via multiplex analyses performed by Eve Technologies (Calgary, AB, Canada). For sample preparation, 1 × 10^7^ FE002-Cart cells were resuspended in 1 mL of PBS and subjected to cell lysis utilizing a freeze–thaw methodology. The crude lysates were subsequently centrifuged at 9000× *g* for 5 min at 4 °C to pellet cellular debris. The total protein concentration within the clarified supernatants was quantified using a standard BCA assay. The resulting supernatants were then subjected to broad-spectrum multiplex profiling using the following specific assay kits: the Human Angiogenesis Array and Growth Factor 17-Plex Array, the Human Cytokine/Chemokine 71-Plex Panel, the Human Soluble Cytokine Receptor 14-Plex Array, the Human MMP and TIMP Panel for Cell Cultures, and the Cytokine TGF-β 3-Plex Array. To ensure accurate comparative analysis, the absolute concentrations of all identified target proteins were subsequently normalized to the total protein content of their respective samples.

#### 2.4.3. Timecourse of HIF-1α Induction with Western Blotting

To evaluate the kinetics of hypoxia-inducible factor (HIF)-1α stabilization, FE002-Cart cells were initially seeded into 6 cm culture dishes in complete growth medium and maintained under standard normoxic conditions until reaching approximately 90% confluence. The cultures were subsequently transferred to controlled hypoxic environments calibrated to 2%, 5%, or 10% O_2_. At predetermined timepoints (0, 3, 6, 8, 24, 48 h), the cells were harvested and subjected to lysis utilizing RIPA buffer supplemented with a comprehensive protease inhibitor cocktail.

Total protein concentrations of the resulting lysates were quantified utilizing a standard BCA assay. Equivalent amounts of total protein were then resolved by sodium dodecyl sulfate-polyacrylamide gel electrophoresis (SDS-PAGE) using NuPAGE™ 4–12% Bis-Tris gradient gels, followed by electrotransfer onto nitrocellulose membranes.

Following appropriate blocking steps, the membranes were probed with primary antibodies directed against either HIF-1α (BD Biosciences, ref. 610958, Allschwil, Switzerland) or a loading control, actin (Thermo Fisher Scientific, ref. PA1-21167, Waltham, MA, USA). Immunoreactive bands were subsequently targeted using the respective horseradish peroxidase (HRP)-conjugated secondary antibodies (anti-mouse-HRP or anti-rabbit-HRP). Final protein visualization was achieved using the ECL™ Prime chemiluminescence detection system (Cytiva, Marlborough, MA, USA).

### 2.5. Cell Spheroid Manufacture Optimization and Functional Validation

#### 2.5.1. Spheroid Formation and Chondrogenic Induction

FE002-Cart cells, previously expanded in 2D cultures, were harvested with TrypLE. The resulting cell suspension was adjusted to a concentration of 1.5 × 10^5^ cells/mL in a chemically defined chondrogenic differentiation medium. To induce spontaneous, scaffold-free, gravity-driven 3D aggregation without centrifugation, 200 µL of the cell suspension (yielding an initial density of 30,000 cells per well) was seeded into 96-well cell-repellent (ultra-low attachment) microplates. The microplates were subsequently statically maintained in a controlled, humidified incubator for a culture duration of up to three weeks. Routine culture maintenance involved partial media exchanges, wherein 50% of the spent medium was carefully replaced with fresh chondrogenic medium three times weekly.

The specific formulation of the chondrogenic differentiation medium was systematically optimized by evaluating the resulting spheroid quality. The primary performance metrics utilized for this evaluation included histological morphology, quantitative GAG content, and the targeted induction of cartilage-specific gene expression (*ACAN*, *COL2*). The iterative compositional modifications (i.e., one single component substituted by a GMP-grade counterpart each time) were rigorously benchmarked against a baseline reference differentiation medium. This baseline medium consisted of DMEM supplemented with 2 mM L-glutamine, 1× penicillin-streptomycin, 1× ITS-II, 100 nM dexamethasone, 10 ng/mL TGF-β3, and 82 µg/mL VitCp. GMP-grade substitutes were ITS X and ITS G (Thermo Fisher Scientific, Waltham, MA, USA) or commercial insulin preparations (Novorapid [Novo Nordisk, Bagsvaerd, Denmark], Fiasp [Novo Nordisk, Bagsvaerd, Denmark], Apidra [Sanofi, Paris, France], Humalog [Eli Lilly, Indianapolis, IN, USA], Lyumjev [Eli Lilly, Indianapolis, IN, USA]) in place of ITS II, Vitamin C Streuli (Streuli, Uznach, Switzerland) in place of VitCp, and Mephameson (Mepha Pharma, Basel, Switzerland), Fortecortin (Merck, Darmstadt, Germany), or Tocris dexamethasone (Tocris Bioscience, Abingdon, UK) in place of research-grade dexamethasone.

#### 2.5.2. CellBrite Cell Staining of Cell Spheroids in Fusion Assays

To track cellular migration and evaluate the dynamics of inter-spheroid fusion, the cells were subjected to vital membrane labeling utilizing CellBrite™ Green and CellBrite™ Red Cytoplasmic Membrane Dyes (Biotium, Fremont, CA, USA). Briefly, prior to the initiation of the fusion assays, the cellular suspensions or intact spheroids were gently washed with PBS and subsequently incubated with the respective CellBrite™ working solutions, prepared in standard culture medium according to the manufacturer’s instructions.

Following a 20 min incubation at 37 °C in the dark to allow for lipophilic dye incorporation into the lipid bilayers, the labeled cells or spheroids underwent three sequential washes with warm complete culture medium. This extensive washing step was critical to ensure the comprehensive removal of any residual, unbound fluorescent probe and prevent non-specific dye transfer.

To assess in vitro structural merging, differentially labeled spheroids (one population uniformly stained green, the other red) were placed into direct physical contact within cell-repellent microplates. The structural integration and cellular mixing between the adjoining spheroids were longitudinally monitored over a 5-day co-culture period. Fluorescent image acquisition and visualization were performed utilizing the IX81 inverted fluorescence microscope (Olympus, Tokyo, Japan) equipped with standard FITC and TRITC/Cy3 optical filter sets to precisely isolate and capture the localized green and red fluorescent signals.

#### 2.5.3. Quantitative Assessment of GAG Synthesis via DMMB Assay

To quantitatively evaluate the deposition of GAGs within the engineered ECM, an analytical 1,9-dimethylmethylene blue (DMMB) assay was performed according to the manufacturer’s specifications. For each experimental condition, spheroids were collected in triplicate samples, with each analytical sample comprising a pool of four individual spheroids. The harvested spheroids were washed once with PBS and subsequently cryopreserved at −80 °C as dry pellets until further processing.

To extract the matrix components, the pooled spheroid samples were subjected to enzymatic digestion in a papain extraction buffer for 3 h, ensuring the complete structural lysis of the 3D constructs. Following this digestion phase, the lysates were centrifuged to pellet any residual insoluble debris, and the clarified supernatants were transferred into fresh microcentrifuge tubes.

The clarified tissue extracts, alongside a defined series of GAG reference standards, were then mixed with the DMMB dye reagent and incubated for 30 min to facilitate the precipitation of the GAG-dye complexes. The samples were centrifuged to isolate the precipitated complexes, and the unbound dye supernatant was carefully discarded to preserve the intact GAG-dye pellet. The dissociation reagent was subsequently added to the pellet, and the mixtures were incubated for 10 min with regular vortexing to ensure the complete dissociation and release of the bound dye.

Following a final centrifugation step to clear the solution, 100 µL aliquots of the dissociated dye from each sample were transferred in duplicate into a 96-well microplate. Absorbance was spectrophotometrically recorded at a wavelength of 656 nm. The total GAG concentration for each sample was interpolated using the generated standard curve, and the data were subsequently normalized to report the mean GAG content per individual spheroid.

#### 2.5.4. Histological Staining and Immunohistochemical Analysis of ECM Components

During the in vitro chondrogenic induction phase, spheroids were harvested and fixed overnight at 4 °C in a 4% neutral buffered formalin solution. Following fixation, the samples were washed three times with PBS and stored in 70% ethanol at 4 °C until processing. To facilitate handling and precise sectioning, the spheroids were pre-embedded in HistoGel™ (Epredia, Portsmouth, NH, USA) prior to routine dehydration and embedding in paraffin wax. Microtome sections of 5 μm thickness were prepared and mounted on glass slides for subsequent analysis.

Following deparaffinization and rehydration, standard histological assessments were performed by blinded operators. General cellular morphology and tissue architecture were visualized using Hematoxylin and Eosin (H&E) staining, while the deposition of sulfated GAGs within the ECM was evaluated using Alcian Blue (AB) staining.

To determine the spatial distribution and expression profiles of specific cartilage matrix proteins, immunohistochemical (IHC) analyses were conducted. Tissue sections were incubated with the following primary antibodies: anti-aggrecan (Thermo Fisher Scientific, ref. AHP0022, Waltham, MA, USA), anti-collagen type II (Abnova, ref. MAB6615, Taipei, Taiwan), anti-collagen type X (Abcam, ref. ab49945, Cambridge, UK), and anti-collagen type I (Abcam, ref. ab138492, Cambridge, UK). Subsequent immunodetection was achieved using the appropriate species-specific ImmPRESS^®^ HRP polymer secondary antibodies (anti-mouse or anti-rabbit, Vector Laboratories, Newark, CA, USA), followed by chromogenic signal development utilizing the ImmPACT^®^ DAB peroxidase substrate system (Vector Laboratories, Newark, CA, USA).

#### 2.5.5. Gene Expression Analysis of Chondrogenic Markers

To evaluate the transcriptional profile and confirm the chondrogenic differentiation of the engineered spheroids, the relative gene expression levels of key matrix components, specifically aggrecan (*ACAN*) and collagen types I (*COL1A1*), II (*COL2A1*), and X (*COL10A1*), were quantified via real-time quantitative PCR (RT-qPCR). At defined timepoints, harvested spheroids were pooled in sterile microcentrifuge tubes and immediately cryopreserved at −80 °C to prevent RNA degradation prior to isolation.

Total RNA was extracted following rigorous mechanical disruption of the pooled spheroids using TRIzol reagent (Thermo Fisher Scientific, Waltham, MA, USA), adhering to the manufacturer’s established protocols. Following RNA quantification, 1 µg of total RNA from each sample was reverse-transcribed into complementary DNA (cDNA) utilizing the PrimeScript™ RT reagent kit (Takara Bio, Shiga, Japan).

Quantitative real-time PCR amplification was subsequently executed in 96-well optical microplates utilizing the KAPA SYBR^®^ FAST qPCR Master Mix (Roche, Basel, Switzerland). All amplification reactions were conducted in technical triplicates to ensure statistical reliability. The relative mRNA expression levels of the target genes (*ACAN* and *COL2A1*) were internally normalized to the constitutive expression of the reference gene *B2M*. Prior to relative quantification, the suitability of *B2M* as a stable reference gene for this specific cellular model was empirically validated. Raw cycle threshold (Ct) values for *B2M* were statistically analyzed across all experimental cohorts (2%, 5%, 10%, and 21% O_2_) and timepoints to ensure that constitutive expression was neither modulated by the depth of hypoxia nor by the 3D differentiation process. Final relative fold changes in gene expression were calculated employing the comparative cycle threshold (2^−ΔΔCt^) method.

The specific forward (F) and reverse (R) primer sequences utilized for the amplifications are detailed in Table 2.

### 2.6. Spheroid Functional and Safety Characterization

#### 2.6.1. Assessment of Cellular Outgrowth and Migration Potential

To evaluate the capacity of FE002-Cart chondroprogenitors to migrate outward from the 3D matrix, a parameter relevant to in vitro microtissue merging, cellular outgrowth assays were performed utilizing spheroids at distinct stages of chondrogenic maturation (specifically, following 1 day, 1 week, and 2 weeks of culture in differentiation medium). Intact spheroids from each predefined maturation timepoint were harvested and individually seeded into standard tissue culture-treated 24-well plates containing complete growth medium to facilitate cell adherence. The kinetics of cellular egress and subsequent 2D radial migration out of the spheroid core were longitudinally monitored via phase-contrast microscopy, with digital microphotographs acquired at predefined time intervals to dynamically document the extent of cellular outgrowth.

#### 2.6.2. Evaluation of Inter-Spheroid Fusion Potential

To serve as modular building blocks, engineered spheroids must possess the intrinsic capacity to structurally merge with adjacent cellular constructs. To assess this autonomous fusion capability, an in vitro structural merging assay was established. Harvested spheroids at distinct stages of chondrogenic maturation (i.e., composed of FE002-Cart cells or adult chondrocytes) were transferred into fresh 96-well cell-repellent microplates containing complete growth medium, facilitating direct physical contact between the constructs without inducing adherence to the well bottom. Furthermore, to determine the functional impact of varying storage and processing conditions on tissue integration, this fusion potential was comparatively evaluated across cohorts of fresh, cryopreserved, and lyophilized spheroids. The morphological progression and overall kinetics of inter-spheroid fusion were longitudinally monitored and documented over time using phase-contrast microscopy.

#### 2.6.3. Assessment of Phenotypic Resilience in Simulated Osteoarthritic Microenvironments

To evaluate the capacity of the engineered spheroids to maintain phenotypic stability and structural integrity when subjected to the catabolic conditions typical of a damaged joint, an in vitro stress assay was conducted. Spheroids were initially cultured for 7 to 14 days in the standard chondrogenic differentiation medium to establish a baseline 3D ECM. Following this initial maturation phase, the constructs were abruptly transitioned into either 100% human osteoarthritic (OA) synovial fluid or a chemically defined degenerative disease (DD) medium for the remainder of a 21-day total culture period.

The simulated DD medium was formulated to specifically replicate a harsh, inflamed joint microenvironment; it consisted of the basal chondrogenic medium supplemented with an acidic and highly pro-inflammatory cocktail comprising 0.45 mg/mL lactic acid (Sigma-Aldrich, Buchs, Switzerland), 100 pg/mL tumor necrosis factor-alpha (TNF-α), 100 pg/mL interleukin-1 beta (IL-1β), and 100 pg/mL interleukin-6 (IL-6, all three from R&D Systems, Minneapolis, MN, USA). Human OA synovial fluid was centrifuged at 3000× *g* for 15 min to remove cellular debris, sterile-filtered, and stored at −80 °C until use.

Following the environmental challenge phase, the spheroids were harvested and comprehensively evaluated to ascertain their functional resilience. The protective capacity of the 3D formulation was quantified by assessing total GAG retention, evaluating overall structural morphology via histological staining, and determining the sustained transcriptional induction of key cartilage-specific genes (*ACAN* and *COL2*) via RT-qPCR.

#### 2.6.4. Evaluation of Spheroid Storage Stability and Logistical Viability

To evaluate logistical feasibility and establish optimal shelf-life parameters for potential off-the-shelf application, the preservation of fresh spheroids under distinct storage conditions was systematically evaluated. Following a standard 2- to 3-week maturation period in chondrogenic differentiation medium, the engineered spheroids were harvested, pooled, and subsequently suspended in defined storage solutions. The cellular constructs were then maintained at either ambient temperature or 4 °C to simulate standard logistical transport and clinical holding environments. In parallel, spheroids were suspended in a cryopreservation solution (DMEM, with 40% FBS, 10% DMSO) and were cryopreserved in liquid nitrogen (freezing curve −1 °C/min).

Following the designated storage intervals, the structural integrity and in vitro biological properties of the spheroids were comprehensively assessed. Cellular viability and metabolic activity were determined utilizing a quantitative MTT (3-(4,5-dimethylthiazol-2-yl)-2,5-diphenyltetrazolium bromide) reduction assay. Briefly, the stored spheroids were incubated in a 5 mg/mL MTT solution for 1 h at 37 °C. The maintenance of cellular metabolic activity was initially confirmed macroscopically by the intracellular reduction in the tetrazolium dye to an insoluble, dark formazan precipitate within the peripheral cell layers. To transition this from a qualitative observation to a rigorous pharmaceutical stability metric, the surrounding MTT solution was carefully aspirated, and the precipitated formazan crystals were completely solubilized through the addition of dimethyl sulfoxide (DMSO) under gentle agitation. The resulting colored solute was transferred to a fresh 96-well microplate (Greiner, Frickenhausen, Germany), and optical absorbance was spectrophotometrically quantified at 570 nm. The relative percentage viability for each storage parameter was subsequently calculated by normalizing these absorbance values against the defined day 0 (unstored) spheroid baseline.

#### 2.6.5. Spheroid Lyophilization and Gamma Irradiation

To evaluate the feasibility of long-term preservation and establish viable off-the-shelf logistical parameters, harvested spheroids were subjected to a controlled lyophilization process, with a subset undergoing subsequent terminal gamma sterilization. Drawing upon previously optimized stabilization protocols for progenitor cell derivatives, the pooled spheroids were resuspended in a defined lyopreservation solution composed of 8% (*m/v*) European Pharmacopoeia (Ph. Eur.)-compliant saccharose and 2% (*m/v*) Ph. Eur.-compliant dextran 40,000 in an aqueous buffer.

The formulated spheroid suspensions were dispensed into nested 2R tubular clear glass lyophilization vials (Schott, Mainz, Germany) and processed utilizing a LyoBeta Mini freeze-dryer (Telstar, Terrassa, Spain). Following an initial freezing phase at −40 °C, primary drying was conducted at −18 °C and 200 mTorr, followed by a secondary drying phase ramped to +20 °C to achieve the final 38 h cycle. Following the completion of drying, the vials were automatically sealed under an argon atmosphere. The resulting lyophilizates were securely boxed and maintained at 4 °C until further processing.

For the gamma-irradiated cohort, the sealed vials containing the lyophilized spheroids were subjected to gamma irradiation utilizing a commercial _60_Co source. The irradiation processing was executed at ambient temperature. To evaluate this preservation method, an exploratory target irradiation dose of 30 ± 5 kGy was applied to the packaged samples. To ensure strict pharmaceutical compliance and validate that the required sterilizing threshold was achieved without exceeding the maximum tolerated exposure, the absolute absorbed radiation dose was analytically confirmed utilizing calibrated alanine-pellet dosimeters placed immediately adjacent to the product vials throughout the irradiation cycle. Following the processing phases, both the non-irradiated (lyophilized only) and gamma-irradiated (lyophilized and sterilized) spheroid cohorts were comprehensively characterized to assess physical stability, matrix retention, and the preservation of inter-spheroid fusion potential.

#### 2.6.6. Assessment of Cellular Safety Attributes in Telomerase Assays

An in vitro telomerase assay was performed using a qPCR telomerase activity quantification kit (ScienCell, Carlsbad, CA, USA) to confirm the non-tumorigenic potential of chondrogenically induced FE002-Cart primary chondroprogenitors following digestion of mature spheroids. Telomerase activity quantification was performed using frozen cellular dry pellets. HeLa and HCT-116 cell lines (ATCC, Manassas, VA, USA) were used as positive controls. Cell lysis was performed using 20 μL of lysis buffer (supplemented with PMSF and β-mercaptoethanol) per million cells before a 30 min incubation phase on ice. The samples were centrifuged at 12,000× *g* at 4 °C for 20 min. For telomerase activity detection, 0.5 μL of sample, 4 μL of 5× telomerase reaction buffer, and 15.5 μL of nuclease-free water were mixed and incubated at 37 °C for 3 h. The reaction was quenched by heating the samples at 85 °C for 10 min. The samples were centrifuged at 1500× *g* for 10 s and stored on ice. The qPCR reactions were prepared in triplicate by mixing 1 μL of the prepared sample, 2 μL of primers, 10 μL of TaqGreen qPCR master mix, and 7 μL of nuclease-free water. The qPCR plates were sealed and centrifuged at 1500× *g* at ambient temperature for 15 s. The qPCR run conditions included an initial denaturation step of 10 min at 37 °C and 36 amplification cycles (i.e., denaturation over 20 s at 95 °C, annealing for over 20 s at 52 °C, and extension for over 45 s at 72 °C). To establish a rigorous analytical baseline, the assay’s limit of detection was defined at a cycle threshold (Ct) value of 33; consequently, any samples exhibiting a Ct value > 33 were strictly classified as negative for telomerase activity. The dynamic range and validity of the assay were confirmed during each run utilizing HeLa and HCT-116 cell lines as highly expressing positive controls. For quantitative comparability, the relative telomerase activity between the pre-formulation 2D cells, the mature 3D payload, and the positive controls was calculated based on the 2^−ΔCt^ method.

### 2.7. Quantitative Morphometry and Image Analysis

To systematically quantify the macroscopic growth kinetics and geometric uniformity of the engineered spheroids, brightfield microphotographs were processed and analyzed utilizing the ImageJ software (v1.54r, National Institutes of Health, Bethesda, MD, USA). Prior to geometric quantification, all acquired images were spatially calibrated against a defined micrometer scale specific to the objective magnification. Raw images were subsequently converted to 8-bit grayscale and subjected to an automated thresholding protocol to generate a binary mask, which precisely delineated the outer boundary of the cellular construct from the surrounding background.

The software’s integrated particle analysis function was then applied to extract the targeted morphometric parameters. The maximum Feret diameter, defined as the longest distance between any two parallel tangents along the construct’s selection boundary, was quantified alongside the cross-sectional area. These continuous morphometric parameters were used to evaluate the overall construct size, calculate the spheroid size distribution across independent biological replicates, and act as a non-destructive proxy for macroscopic tissue assembly. Concurrently, the construct circularity index was calculated utilizing the formula

4 × *π* × (*Area*/*Perimeter*^2^)

to quantitatively assess the structural consolidation and geometric homogeneity of the microtissues across different manufacturing batches, where a maximum value of 1.0 represents a mathematically perfect circle.

### 2.8. Statistical Analysis and Data Presentation

Statistical analyses were systematically conducted to evaluate the significance of differences between experimental conditions. All quantitative data are expressed as the mean ± standard deviation of at least three independent biological or technical replicates, unless otherwise specified. For the purposes of this study, a biological replicate was defined as a completely independent spheroid manufacturing batch generated from a newly thawed cell vial. Conversely, technical replicates consisted of multiple individual spheroids sampled from within the same manufacturing batch. Prior to any statistical testing, all technical replicates within a single manufacturing batch were averaged to yield a single, independent data point representing that batch. Furthermore, as the FE002-Cart working cell bank replicates originate from a single characterized donor bank, statistical variances reported for this specific cohort represent independent manufacturing run (batch-to-batch) variability rather than true inter-donor “biological variability”.

Furthermore, to minimize positional bias and edge effects during in vitro functional and stress evaluations, edge rows/columns were filled with blank medium (no spheroids seeded) and sample placements within multi-well microplates were systematically randomized prior to data acquisition. Prior to comparative analysis, the normality of the datasets was rigorously verified, utilizing the Shapiro–Wilk test to assess normal distribution and Levene’s test to evaluate the homogeneity of variances across groups. To compare the means of two paired datasets, a paired Student’s *t*-test was employed, while an unpaired Student’s *t*-test was utilized for independent two-group comparisons. In instances where datasets failed to meet the assumptions of normality, the non-parametric Mann–Whitney U test was applied.

For the evaluation of a single independent variable across three or more groups, a one-way analysis of variance (ANOVA) was conducted. To appropriately account for the nested structure of the comparative data between the single allogeneic FE002-Cart cell type and the multiple primary adult chondrocyte types, a linear mixed-effects model was utilized. In this model, ‘donor’ was strictly designated as a random effect to mathematically de-confound inter-donor biological variability (patient-to-patient) from intra-donor manufacturing variance (batch-to-batch). Furthermore, multifactorial datasets, such as those assessing the combinatorial effects of varying oxygen levels and dexamethasone concentrations, were analyzed using a two-way ANOVA to identify both main and interaction effects. To appropriately address the correlated nature of longitudinal data, such as sequential GAG accumulation, cellular proliferation kinetics, or chondrogenic gene induction over the culture period, a repeated-measures ANOVA was performed.

Following statistically significant findings in the primary ANOVA models, post hoc multiple comparison analyses were conducted using Tukey’s Honestly Significant Difference (HSD) test or Bonferroni corrections to ascertain specific group-level differences. The threshold for statistical significance was defined a priori as a *p*-value < 0.05. Furthermore, to rigorously convey the biological magnitude of observed phenomena, effect sizes were contextually reported throughout the study as absolute mean differences, relative percentage changes, or transcriptomic fold inductions where feasible. Finally, all statistical computations, data processing, and graphical representations were executed utilizing Microsoft Excel and Microsoft PowerPoint (Microsoft Corporation, Redmond, WA, USA), alongside the GraphPad Prism software, version 8.0.2 (GraphPad Software, San Diego, CA, USA).

## 3. Results

### 3.1. Defining Microenvironmental Formulation Parameters for 3D Chondroprogenitor Microtissues: Hypoxia and Glucocorticoid Thresholds

In the development of cell-based cartilage ATMPs, the spatial organization of the cellular active substance and the biochemical constituents of the differentiation medium must be rigorously controlled as critical formulation parameters. To effectively engineer a functional cartilage substitute and prevent the spontaneous cellular dedifferentiation commonly observed in 2D expansion, we investigated how strict microenvironmental control drives the phenotypic stability and ECM payload of a 3D FE002-Cart formulation.

**Figure 1:**
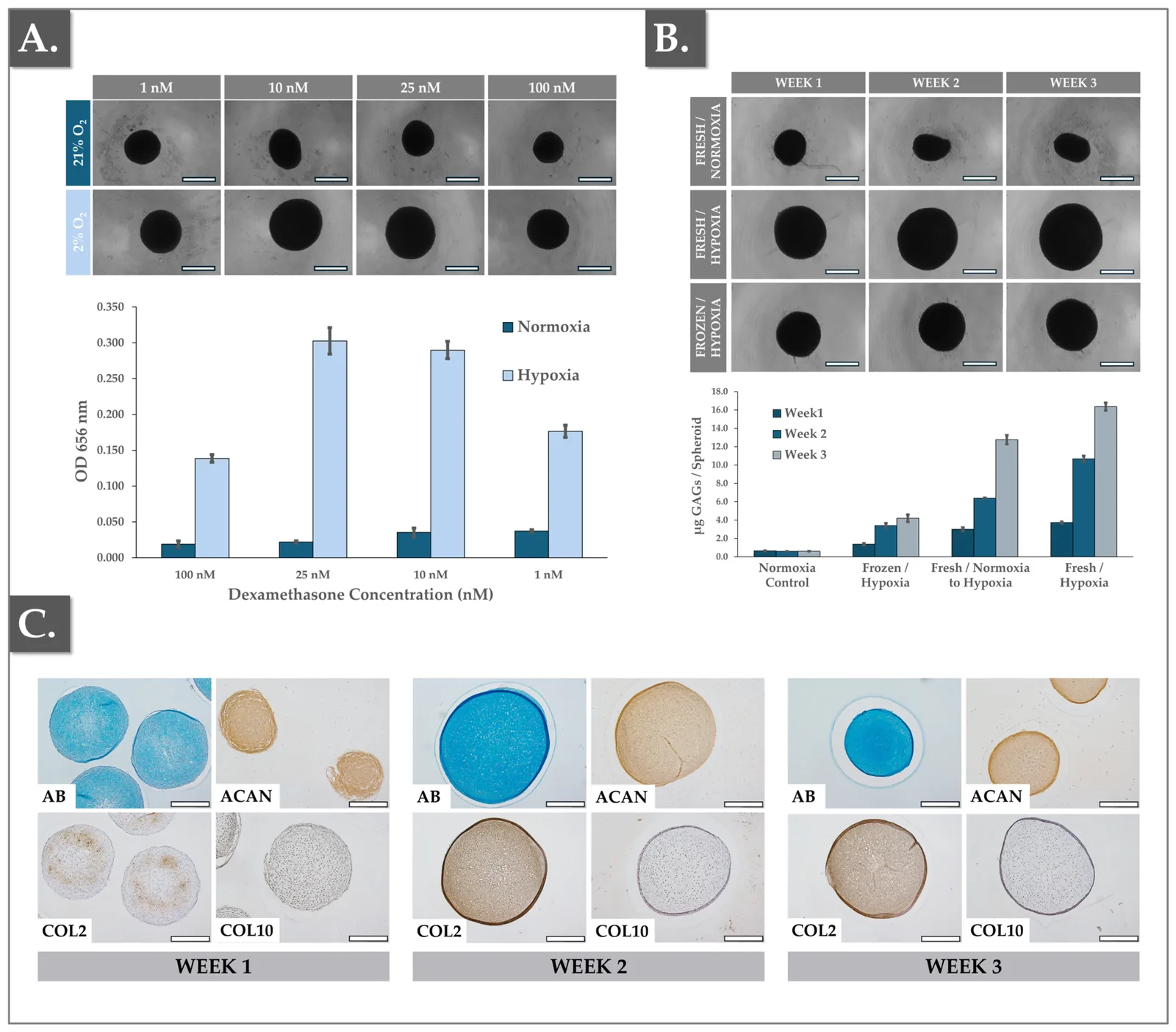
Oxygen tension, dexamethasone concentration, and cell source dictate FE002-Cart spheroid development, GAG accumulation, and chondrogenic differentiation. (**A**) Impact of oxygen and dexamethasone on spheroid growth and GAG production. FE002-Cart spheroids were cultured for three weeks in chondrogenic medium supplemented with varying concentrations of dexamethasone under hypoxic (2% O_2_) or normoxic (21% O_2_) conditions. Representative macroscopic images of the spheroids at week 3 alongside the corresponding quantification of GAG content. (**B**) Effects of oxygen level and cell source type on spheroid development. Spheroids were generated either from freshly expanded 2D monolayers or directly from cryopreserved cell stocks, then cultured in chondrogenic medium containing 10 nM dexamethasone. Spheroid morphology and GAG content were monitored weekly over a three-week period. Progressive increases in spheroid size and GAG accumulation were observed exclusively in the hypoxic environment. Furthermore, spheroids derived from fresh 2D cells exhibited enhanced morphological development and GAG production compared to those originating from frozen stocks. (**C**) Chondrogenic marker accumulation over time. Representative histological cross-sections of spheroids cultured under hypoxic (2% O_2_) conditions at weeks 1, 2, and 3. Sections illustrate progressive ECM deposition and chondrogenic differentiation, demonstrated through Alcian Blue (AB) staining and immunohistochemistry for aggrecan (ACAN), type II collagen (COL2), and type X collagen (COL10). Scale bars = 250 μm. Quantitative data are presented as the mean ± standard deviation (SD) of *n* = 3 independent manufacturing batches, with each analyzed data point representing the pooled average of technical replicates within that respective batch. Statistical significance (*p* < 0.05) was determined using a one-way ANOVA followed by Tukey’s HSD post hoc test for multiple comparisons.

Initial characterizations of the formulation process revealed that the synthetic glucocorticoid concentration acts as a master threshold for 3D matrix assembly. By evaluating distinct dexamethasone concentrations under strictly controlled gas environments, it was established that the commonly utilized high-dose exposure (100 nM), as well as sub-therapeutic limits (1 nM), are in fact actively detrimental to the biological product, resulting in macroscopically stunted spheroids and diminished GAG content (p < 0.01 compared to the optimal 10 nM concentration, via one-way ANOVA with Tukey’s post hoc correction, n=3 independent batches; Figure 1A).

The optimal biochemical window for robust formulation was strictly defined between 10 and 25 nM dexamethasone. To maximize specific chondrogenic signaling while limiting potential off-target excipient toxicity, 10 nM dexamethasone was validated as the standard biochemical parameter for all subsequent 3D microtissue manufacturing in the study.

The maturation kinetics of the 3D formulation were subsequently defined to establish precise manufacturing timelines. Spheroid size and total GAG content exhibited a continuous, linear increase throughout a 3-week 3D culture period, validating active and sustained matrix accumulation (Figure 1B). Histological assessment demonstrated that initial collagen type II (COL2) deposition occurred by week 1, progressing into a highly homogeneous structural network by week 2, thereby establishing a mature cartilage-like matrix payload (Figure 1C). Crucially, the data indicated that pre-conditioning the cells through a 2D expansion phase prior to 3D formulation yielded significantly higher GAG production compared to spheroids generated directly from frozen stocks (p < 0.01, via an unpaired Student’s *t*-test, n=3 independent biological replicates), confirming 2D pre-culture as a mandatory preparative step for this ATMP prototype.

**Figure 2:**
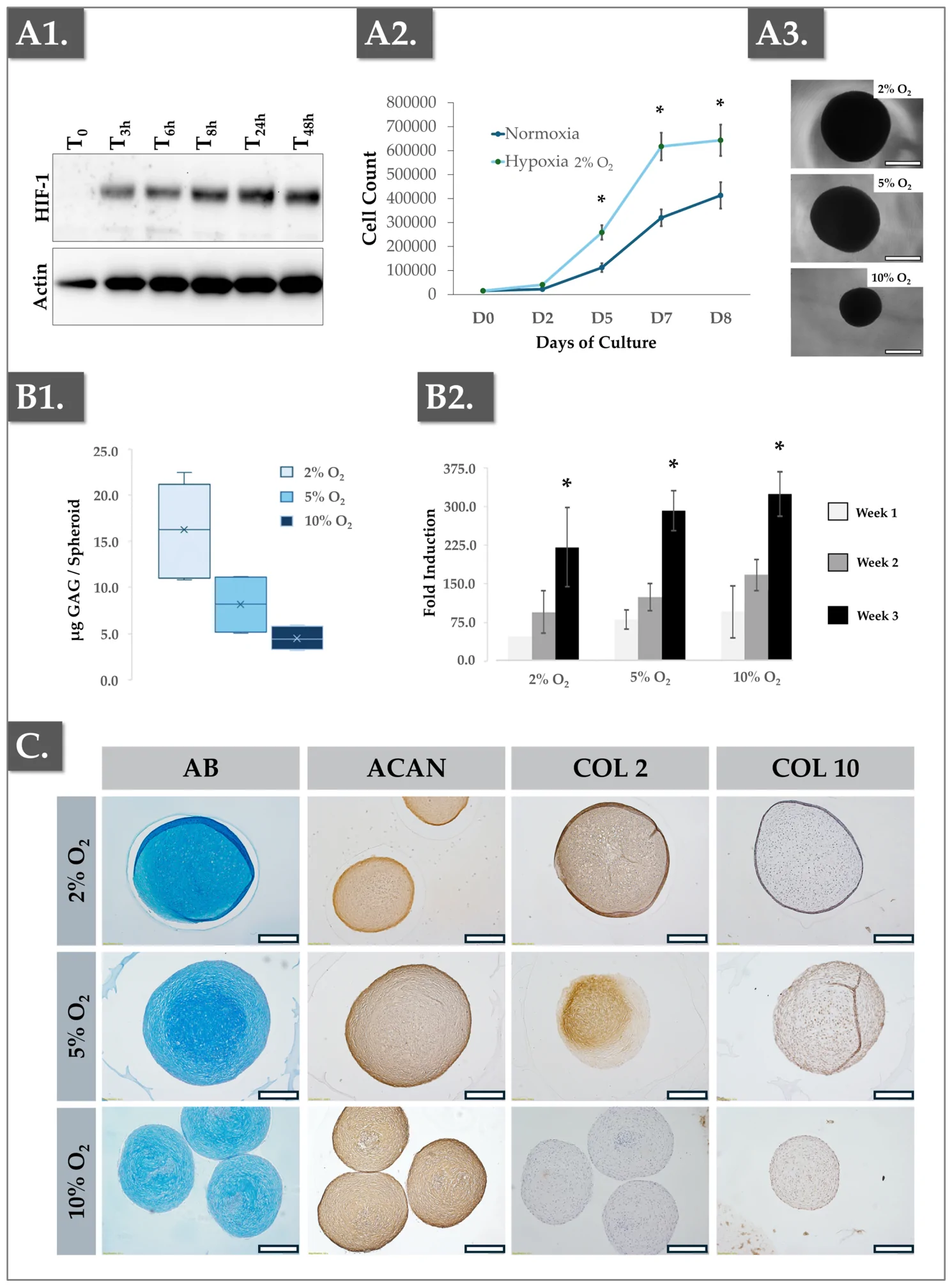
Effect of oxygen tension on 2D cell expansion and 3D FE002-Cart spheroid development. (**A1**) Representative Western blot demonstrating robust HIF-1 induction over time following the transfer of cells from normoxia to a 2% O_2_ culture condition. Actin was utilized as a loading control. (**A2**) Comparison of 2D cell proliferation curves in 2% hypoxia versus normoxia. Culturing in 2% hypoxia resulted in a statistically significant increase in overall cell proliferation. (**A3**) Representative macroscopic images of 3-week spheroids cultured in 2%, 5%, and 10% O_2_, demonstrating that macroscopic spheroid size and formation are visibly impacted by varying O_2_ concentrations. (**B1**) Biochemical quantification of GAGs at 3 weeks of culture. GAG accumulation was highest in the 2% O_2_ condition, which directly correlates with the increased spheroid size. (**B2**) Progressive *ACAN* fold induction over a 3-week culture period across the distinct oxygen concentrations, as measured by real-time PCR and relative to Day 0 values. (**C**) Representative histological evaluation of 3-week spheroids cultured under 2%, 5%, and 10% O_2_. The panels display ECM composition and distribution through Alcian Blue (AB) staining, alongside immunohistochemical profiling for aggrecan (ACAN), type II collagen (COL2), and type X collagen (COL10). Scale bars = 250 μm. Quantitative data are presented as the mean ± standard deviation (SD) of *n* = 3 independent manufacturing batches, with each analyzed data point representing the pooled average of technical replicates within that respective batch. Statistical significance was indicated by an asterisk *.

Furthermore, because native articular chondrocytes inhabit a stratified hypoxic niche in vivo (ranging from 5–10% O_2_ in superficial zones to approximately 1% in deep zones), we treated ambient oxygen tension not merely as a culture condition, but as a critical active process parameter for 3D microtissue formulation. The cellular response to three distinct physiological hypoxic environments (2%, 5%, and 10% O_2_) was rigorously quantified (Figure 2).

During the initial 2D expansion phase, hypoxic incubation significantly accelerated cellular proliferation across all tested parameters (2%, 5%, and 10% O_2_) compared to normoxic controls (*, p < 0.05, via repeated-measures ANOVA with Bonferroni correction, n=3 biological replicates; Figure 2(A2)). However, the stabilization of the fundamental hypoxia-inducible factor (HIF)-1 signaling pathway was strictly dependent on the depth of the applied hypoxia. Western blot analysis revealed that exposure to 2% and 5% O_2_ triggered a rapid, robust, and sustained accumulation of HIF-1α protein, with strong expression detectable within 3 h and persisting beyond 24 h under 2% O_2_ (consistently observed across *n* = 3 independent cell expansions and protein extractions; Figure 2(A1)). Conversely, the 10% O_2_ environment proved insufficient to mount a robust molecular response, yielding a severely blunted, barely detectable stabilization of HIF-1α over the same time course.

Overall, the transition to the 3D spheroid formulation revealed high sensitivity to absolute oxygen tension, directly dictating the quality of the final therapeutic product. Macroscopically, deeper hypoxia (2% and 5% O_2_) resulted in significantly larger spheroids (*p* < 0.001) compared to those manufactured at 10% O_2_ or in standard normoxia (Figure 2(A3)). Quantitative image analysis confirmed that the maximum construct cross-section (Feret diameter) was strictly and inversely proportional to oxygen concentration: measuring 1.28 ± 0.06 mm at 2% O_2_, 1.11 ± 0.13 mm at 5% O_2_, 0.96 ± 0.14 mm at 10% O_2_, and 0.75 ± 0.02 mm under normoxia (all conditions demonstrating highly significant reductions when compared to 2% O_2_, *p* < 0.001, via one-way ANOVA with Tukey’s post hoc correction). Furthermore, morphological assessment revealed subtle variations in construct circularity. While spheroids cultured at 5% O_2_ (0.60 ± 0.09) and normoxia (0.53 ± 0.07) did not significantly differ in shape from the optimal 2% O_2_ condition (0.57 ± 0.05), those grown at 10% O_2_ exhibited a statistically higher circularity index (0.68 ± 0.06, *p* < 0.001, via one-way ANOVA with Tukey’s post hoc correction, n=3 independent batches). Ultimately, the volumetric growth driven by deep hypoxia translated into stark differentials in the biochemical payload, as the mean GAG content reached 16.37 ± 5.2 µg per spheroid at 2% O_2_, compared to 7.49 ± 3.7 µg at 5% O_2_, and a severely stunted 3.47 ± 1.5 µg at 10% O_2_ (Figure 2(B1)).

This microenvironmental dependency was validated at the molecular level, where quantitative PCR revealed significant differences in the magnitude of gene induction. While robust transcriptional activation of both *ACAN* and *COL2* occurred at 2% and 5% O_2_, this response was severely blunted at 10% O_2_. Under optimal hypoxia (2% and 5%), *ACAN* expression amplified progressively over the three-week period. By week 3, *ACAN* induction culminated in remarkable 224-fold and 309-fold increases for the 2% and 5% O_2_ conditions, respectively (*, p < 0.001 relative to Day 0, via repeated-measures ANOVA, n=3 biological replicates; Figure 2(B2)). In parallel, week 3 *COL2* induction reached 1.68 × 10^6^-fold and 0.64 × 10^6^-fold increases for the 2% and 5% O_2_ conditions, respectively (p < 0.001 relative to Day 0, via repeated-measures ANOVA, n=3 biological replicates).

Crucially, while *ACAN* transcriptional induction peaked under higher investigated O_2_ conditions, the ultimate definition of the critical process parameters (CPP) prioritized the functional translation and accumulation of the ECM over transient mRNA expression levels. Furthermore, a two-way ANOVA confirmed a highly significant interaction effect between specific oxygen tensions and dexamethasone concentrations in driving optimal macroscopic growth and matrix synthesis, statistically validating the synergistic necessity of combining 2% O_2_ with 10 nM dexamethasone.

Consequently, to ensure the phenotypic stability and maximum functional payload of the formulated prototype, the CPP for manufacturing FE002-Cart 3D microtissues dictate an initial hypoxic 2D expansion, followed by 3 weeks of 3D differentiation maintained at 2% O_2_ utilizing 10 nM dexamethasone. A comprehensive scoring of these selection criteria across the varying oxygen tensions is detailed in Table 3.

Furthermore, while deep hypoxia drastically altered 3D matrix assembly, proteomic characterizations confirmed that hypoxic incubation only subtly modulated the baseline soluble protein profile of the 2D expanded cells prior to formulation (Table 4).

However, a targeted analysis of the full proteomic dataset revealed critical functional validations of the active HIF-1 signaling pathway. Specifically, the potent angiogenic factors VEGF-A and VEGF-C, classic downstream targets of HIF-1α transcriptional activity, were upregulated by hypoxia. In detail, VEGF-A expression increased proportionally with the depth of hypoxia, exhibiting significantly elevated (*p* < 0.01, via one-way ANOVA with Tukey’s post hoc correction, *n* = 3 biological replicates) concentrations at both 2% O_2_ (199.7 ± 26.4 pg/mg) and 5% O_2_ (156.8 ± 11.9 pg/mg) compared to 10% O_2_ (92.4 ± 7.2 pg/mg) and normoxic (99.5 ± 12.0 pg/mg) controls. Concurrently, VEGF-C demonstrated a highly specific > 3-fold upregulation exclusively under the optimal 2% O_2_ condition (300.6 ± 31.7 pg/mg) relative to 5% O_2_ (96.5 ± 29.1 pg/mg), 10% O_2_ (63.5 ± 8.1 pg/mg), and normoxia (94.8 ± 10.2 pg/mg). This confirmed that the robust HIF-1α stabilization observed at 2% O_2_ translates directly into functional downstream molecular cascades prior to 3D tissue assembly.

### 3.2. Benchmarking Engineered Microtissue Consistency: Overcoming Adult Chondrocyte Donor Variability

A fundamental bottleneck in the clinical translation of autologous cartilage therapies is the inherent biological variability of patient-derived cells. Rather than serving as a direct statistical comparison of therapeutic efficacy, the 3D formulation profiles of primary adult human articular chondrocytes derived from seven independent donors were evaluated to illustrate the inherent biological unpredictability of autologous platforms. The inter-donor variability of these adult cells was contrasted against the intra-donor manufacturing consistency of the banked allogeneic FE002-Cart cell source to highlight the lot-to-lot reliability required for standardized 3D microtissue production.

#### 3.2.1. Morphological Heterogeneity and Structural Consolidation

All primary cell types were subjected to the stringently optimized engineered microtissue manufacturing parameters (2D hypoxic pre-conditioning followed by 3D scaffold-free aggregation at 2% O_2_ with 10 nM dexamethasone). Under these defined conditions, the adult primary chondrocytes from seven adult donors exhibited profound inter-donor heterogeneity. While the FE002-Cart cells rapidly and reproducibly self-assembled into spherical, tightly cohesive microtissues with highly uniform Feret diameters across all biological replicates, the adult chondrocyte cohorts were morphologically erratic (Table 5).

Depending heavily on the donor, adult cells frequently formed asymmetrical, loosely aggregated constructs, or exhibited highly unpredictable macroscopic diameters. For instance, while certain adult donor constructs achieved robust spherical consolidation, others remained drastically smaller and morphologically stunted under the exact same manufacturing parameters.

#### 3.2.2. Biochemical and Transcriptional Unpredictability

This morphological inconsistency was directly mirrored in the functional ECM payload. Utilizing the linear mixed-effects model to control for ‘donor’ as a random effect, quantitative analysis of the 3D constructs revealed massive deviations in both the mean GAG deposition and the intra-donor variability among the different primary adult chondrocyte cohorts, distinctly separating their unpredictable output from the highly consistent baseline of the FE002-Cart batches. For example, while donor E yielded exceptionally high matrix deposition (46 ± 5 µg GAGs/spheroid), other donors exhibited significantly impaired functional outputs with wide variability, with donor C producing roughly 12 ± 2 µg and donor F yielding a severely depressed output of 9 ± 2 µg of GAGs per spheroid (Table 5). Whereas the FE002-Cart cells demonstrated a robust, predictable, and uniform accumulation of GAGs across manufacturing batches, these adult-derived spheroids exhibited erratic and highly unpredictable matrix yields.

Transcriptional profiling via RT-qPCR further unmasked the phenotypic instability of the adult cells. While FE002-Cart spheroids consistently exhibited targeted and exponential inductions of essential hyaline cartilage markers (*ACAN* and *COL2*), the adult chondrocyte cohorts displayed highly variable transcriptomic responses. Crucially, even under strict hypoxic control (2% O_2_), several adult donor cell types demonstrated an unwanted upregulation of *COL1A1* (indicative of fibrocartilage formation) or *COL10A1* (a marker of terminal hypertrophic degradation, Table 5). This indicates a strong propensity for phenotypic drift that is extremely difficult to override using solely environmental and biochemical cues.

#### 3.2.3. Implications for Clinical Manufacturing

The stark biological unpredictability observed in primary adult chondrocytes fundamentally limits their utility for standardized, large-scale 3D biomanufacturing. The inability to guarantee a uniform therapeutic payload or stable phenotype from batch to batch poses a severe regulatory and clinical risk. By contrast, these data validate the use of the banked, highly characterized allogeneic FE002-Cart cell type. Utilizing this standardized clinical-grade source effectively circumvents patient-to-patient biological variability, providing the rigorous lot-to-lot phenotypic consistency and structural predictability that are prerequisites for commercial ATMP manufacturing.

### 3.3. Transitioning to GMP-Compatible Manufacturing: Defining Biological Sensitivities to Pharmaceutical Excipients

Having established the standardized FE002-Cart cell type as a reliable biological starting material to overcome autologous donor variability, the next critical translational hurdle was securing the biochemical manufacturing process. To evaluate the biological impact of transitioning to a clinical-scale manufacturing process, research-grade reagents were systematically substituted with Good Manufacturing Practice (GMP)-compatible equivalents. We evaluated the biological impact of this raw-material transition on the morphological development, GAG content, and chondrogenic induction (*ACAN* and *COL2*) of the 3D engineered microtissues.

Initial formulation optimization focused on the insulin-transferrin-selenium (ITS) supplement. Upon evaluating multiple certified ITS formulations and five distinct commercial insulin sources, it was determined that the complex ITS supplement could be effectively simplified and substituted with pharmaceutical-grade insulin alone (e.g., Humalog or Novorapid, Table 6).

This substitution significantly streamlined the final engineered microtissue formulation without compromising the necessary metabolic support for matrix assembly. Similarly, the research-grade ascorbic acid source was successfully replaced with an approved commercial vitamin C formulation (Vitamin C Streuli, Table 6).

The most profound biological sensitivity, however, was unmasked during the substitution of the master differentiation regulator, dexamethasone. While tightly controlled dexamethasone levels are strictly required for robust chondrogenesis, integrating two standard pharmaceutical-grade commercial formulations (Fortecortin and Mephameson) resulted in severely compromised 3D microtissue quality (Table 6). Macroscopic evaluation revealed distinctly divergent, yet uniformly detrimental, morphological responses. Mephameson led to severely stunted and non-homogenous spheroid development, with mean spheroid size reductions of 52 ± 4% (p < 0.001, via one-way ANOVA with Tukey’s post hoc correction, n=3 independent batches) as compared to historical baselines. Fortecortin, on the other hand, yielded a more homogenous structural formation, but the resulting constructs remained substantially smaller (mean size reductions of 36 ± 2%, p < 0.001, n=3) and visibly stunted compared to those produced with the research-grade standard. Furthermore, these clinical-grade sources resulted in significantly depressed GAG deposition and impaired *COL2* induction. This critical finding indicates that undefined excipients or preservatives present in standard commercial drug formulations can actively interfere with and abort the chondrogenic differentiation program in 3D culture. Consequently, a highly purified, ancillary-grade dexamethasone (Tocris) was identified and validated as a biologically compatible necessity; spheroids manufactured with the Tocris dexamethasone successfully restored the robust macroscopic size and spherical morphology to completely match the historical control.

Following the rigorous independent qualification of each individual component, the combined GMP-compliant differentiation medium formulation was finally evaluated against the baseline research medium. The final engineered clinical-grade formulation consisted of basal DMEM supplemented with 2 mM L-glutamine, 10 µg/mL pharmaceutical insulin (Novorapid, Novo Nordisk), 10 nM ancillary-grade dexamethasone (Tocris), 10 ng/mL animal-free TGF-β3, and 50 µg/mL clinical-grade vitamin C (Streuli). Spheroids manufactured utilizing this fully defined, GMP-compliant medium exhibited morphological growth kinetics, Feret diameters, and absolute GAG contents that were comparable to those produced with the research-grade medium, with no statistically significant differences detected between the formulations (*p* > 0.05, via an unpaired Student’s *t*-test, *n* = 3 independent batches).

Finally, transcriptional profiling confirmed that spheroids manufactured with the fully defined, GMP-compliant medium maintained comparable inductions of *ACAN* and *COL2* relative to the historical controls, similarly exhibiting no statistically significant differences (*p* > 0.05, via an unpaired Student’s *t*-test, *n* = 3 independent batches). Together, these biochemical, morphological, and transcriptional results highlight that rigorous raw-material qualification is a critical requirement in ATMP manufacturing. Careful selection of GMP-grade components ensures that clinical excipients do not compromise the phenotypic identity, matrix quality, and structural integrity of the final therapeutic product.

### 3.4. Characterization of the 3D Engineered Microtissue Formulation: Phenotypic Resilience and Tissue Integration Kinetics

To successfully translate a living cellular therapy to the clinic, an engineered product must not only demonstrate baseline functionality but also survive simulated catabolic microenvironments. Consequently, we rigorously characterized the phenotypic resilience, protective capacity, and the in vitro tissue merging properties of the formulated spheroids.

#### 3.4.1. Phenotypic Resilience in Simulated Osteoarthritic Microenvironments

Following in vivo administration, the cellular payload is inevitably exposed to pathological, inflamed, and acidic tissue conditions. To evaluate the protective capacity of the 3D formulation, week 2 (W2) spheroids were subjected to a 7-day challenge in a degenerative disease (DD) medium. Crucially, the 3D microtissue formulation effectively shielded the cellular payload, with all spheroids retaining robust metabolic viability (confirmed via positive MTT staining) under both strictly acidic and combined inflammatory conditions. While exposure to the complete DD medium or the isolated cytokine cocktail induced a statistically significant depletion in total GAG content (p < 0.01*,* via a repeated-measures ANOVA with Bonferroni correction, n=3 independent biological replicates, dropping from 21 ± 2 µg/spheroid in the positive control to 12 ± 1 µg and 13 ± 2 µg, respectively), the acidic environment alone (lactic acid) did not significantly compromise GAG levels, maintaining a robust yield of 20 ± 2 µg/spheroid (p > 0.05 compared to the positive control, via a one-way ANOVA, n=3 biological replicates). This quantitatively confirmed that the negative impact on the matrix is primarily mediated by the inflammatory cytokine cocktail rather than the acidic pH of the diseased joint. Furthermore, histological evaluations (Alcian Blue and aggrecan staining) revealed no obvious structural degradation or architectural differences across the experimental groups, underscoring the structural stability of the matrix.

To approximate the clinical administration site even more closely, W2 spheroids were directly exposed to 100% grade IV OA human synovial fluid for 7 days. The spheroids maintained excellent cellular viability. Notably, spheroids transitioned to an unsupplemented basal DMEM control exhibited severe GAG depletion (lowering to approximately 3.5 µg/spheroid; *p* < 0.001 compared to day 0 baseline, via a one-way ANOVA, *n* = 3 biological replicates) and rapid physical shrinkage. In stark contrast, constructs maintained across three distinct 100% OA synovial fluid donor samples (SF1, SF2, and SF3) exhibited comparatively low GAG loss (retaining 12 ± 3, 14 ± 2, and 14 ± 3 µg/spheroid, respectively, across n=3 technical replicates per fluid donor) and maintained their structural volume without shrinking. These data confirm that the robust 3D ECM acts as a physical shield, rendering the formulated spheroids highly compatible with pathological synovial fluids and capable of resisting rapid degradation.

#### 3.4.2. In Vitro Structural Integration: Cellular Egress and Inter-Spheroid Fusion Potential

**Figure 3:**
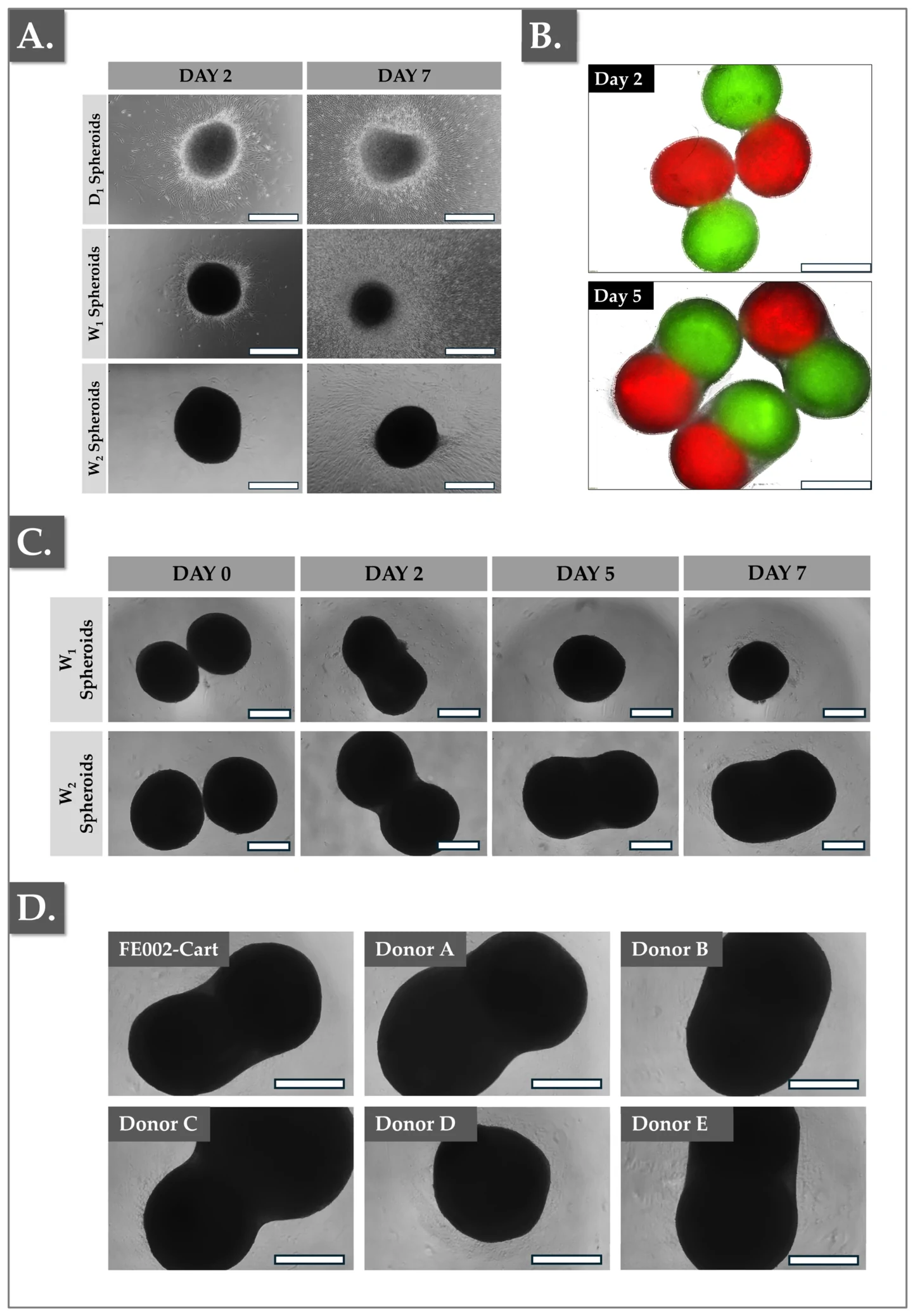
In vitro structural merging, cellular migration, and inter-spheroid fusion potential of engineered FE002-Cart microtissues. (**A**) To evaluate the capacity for cellular egress, cellular migration out of the spheroids was assessed following their previous maturation over 1 day (D_1_), 1 week (W_1_), or 2 weeks (W_2_) in chondrogenic medium. The kinetics of cellular egress and 2D radial migration were monitored at 2 and 7 days post-seeding, demonstrating that migration potential inversely correlates with the maturation stage of the construct. Cellular outgrowth was progressively reduced between D_1_, W_1_, and W_2_ spheroids. (**B**) To dynamically visualize structural integration, FE002-Cart cells were labeled with either red or green fluorescent CellBrite dyes prior to spheroid formation and differentiated for two weeks. Spheroid fusion was monitored longitudinally at days 2 and 5. Initial spheroid connection is observed already at day 2, and while boundary integration is further increased at day 5, where the distinct internal cores of the two merging spheroids remain clearly visible. (**C**) Brightfield macroscopic evaluation of inter-spheroid fusion utilizing spheroids matured for 1 week or 2 weeks in chondrogenic medium. Consistent with the cellular migration data, the autonomous fusion potential progressively decreases with increased spheroid maturation. (**D**) Evaluation of the heterologous fusion potential of fetal spheroids with adult spheroids. FE002-Cart spheroids were placed in direct physical contact with either an identical spheroid as a baseline control or with spheroids produced from different adult donor chondrocytes. Representative macroscopic integration and tissue merging were monitored and documented after nine days of co-culture. Scale bars = 500 μm. Representative macroscopic and fluorescent images were acquired from n=3 independent biological experiments to ensure reproducibility.

To evaluate the modular capacity of the engineered microtissues, we assessed their in vitro structural merging potential by monitoring cellular outgrowth and inter-spheroid fusion at different maturation stages (Figure 3).

Cellular migration out of the 3D construct was found to be inversely proportional to the maturation time of the spheroid, likely governed by the increasing density of the synthesized ECM. Day 1 (D1) cellular aggregates exhibited rapid cellular egress. Conversely, the outgrowth kinetics were progressively delayed as the constructs matured, quantified sequentially as D1 > W1 > W2 (observations consistent across *n* = 3 independent maturation batches; Figure 3A). This inverse correlation suggests that the release kinetics of the cellular payload into the defect site can be tuned based on the manufacturing maturation time.

Similarly, the capacity for modular tissue assembly was confirmed via inter-spheroid fusion assays. While fusion initiation was successful across various maturation stages, the speed and completeness of the structural integration were influenced by construct maturity. Utilizing a 5-day CellBrite fluorescent tracking assay, W2 spheroids labeled green and red demonstrated robust physical connection and initial boundary integration. However, the denser matrix delayed the complete fusion of their internal cores within the isolated in vitro setup (Figure 3B). W1 spheroids exhibited rapid and advanced fusion, merging into a unified, rounder macroscopic structure (Figure 3C). Importantly, engineered FE002-Cart spheroids successfully initiated fusion with spheroids derived from various adult primary chondrocytes. While the degree of complete structural integration (spherical remodeling) exhibited donor-dependent variability, the data demonstrated that these constructs possess the intrinsic and versatile capacity to structurally merge with adjacent heterologous microtissues in vitro (Figure 3D).

#### 3.4.3. Payload Viability and In Vitro Telomerase Activity Assessment

Beyond confirming the macroscopic capacity for structural host tissue integration, it was imperative to validate the intrinsic health and biosafety profile of the cellular payload following extended 3D encapsulation. To definitively validate the integrity of the cellular payload within the mature formulation, 3-week spheroids were subjected to overnight enzymatic digestion with collagenase II. The recovered payload exhibited excellent health, maintaining > 90% cellular viability across *n* = 3 independent digestion assays. Furthermore, when replated into standard 2D culture, these recovered cells rapidly adhered, exhibited their characteristic morphology, and fully resumed proliferation, confirming that deep 3D matrix encapsulation does not induce irreversible senescence.

From a biosafety perspective, it was critical to ensure that the extended 3D manufacturing process did not inadvertently trigger oncogenic pathways. Telomerase activity levels in the cells post-3D maturation remained completely undetectable, exhibiting Ct values well beyond the established threshold value of 33. This stood in stark contrast to the robust amplification observed in the tumorigenic positive controls (HeLa and HCT-116 cell lines), which reliably amplified at average Ct values of 16.5 ± 0.8 and 17.2 ± 0.5, respectively (*n* = 3 technical replicates per cell line). Furthermore, the negative expression profile of the recovered 3D payload was quantitatively identical to the baseline of the same cells in their pre-formulation 2D state. This definitive lack of telomerase activation provides quantitative confirmation that the 3D manufacturing process does not trigger apparent cellular transformation, providing preliminary in vitro data supporting a lack of cellular transformation.

### 3.5. Establishing Logistical Viability: Storage Parameters for Engineered Microtissues

While the dense 3D ECM successfully shields the cellular payload from extreme in vivo catabolic conditions, these biological advantages are rendered clinically irrelevant if the formulated microtissues cannot survive the logistical supply chain prior to surgical implantation. The clinical utility and commercial scalability of living cellular therapies are frequently restricted by rigid cold-chain logistics and prohibitively short product shelf-lives. To establish the practical clinical applicability of the 3D FE002-Cart formulation, we systematically characterized optimal pre-implantation storage/transport conditions and defined the logistical shelf-life of the final product. The efficacy of the storage parameters was rigorously evaluated based on the retention of cellular metabolic activity (via MTT reduction), the preservation of total GAG matrix content, and the maintenance of essential inter-spheroid fusion capacity.

Initial assessments of short-term liquid storage revealed that standard clinical transport solutions, including 0.9% NaCl, Ringer’s solution, PBS, DPBS, and 5% glucose, were entirely insufficient to support the metabolic demands of the dense 3D constructs, resulting in a rapid failure to maintain spheroid viability (indicated by a completely negative MTT signal, Table 7).

Notably, even supplementing these clinical solutions with 2% human serum albumin (HSA) failed to rescue cellular metabolic activity. In stark contrast, suspension in a minimal basal medium (DMEM, high glucose, with sodium pyruvate, without phenol red) was the only tested condition that successfully preserved the functional and structural integrity of the engineered microtissue.

Crucially, while conventional cellular biologics typically mandate strict refrigeration, systematic evaluation at 2, 4, and 7 days post-harvest revealed that cold storage actively impaired the engineered microtissues. Specifically, spheroids maintained under standard refrigerated conditions (4 °C) rapidly exhibited compromised metabolic viability (assessed via MTT) and a severely impaired capacity for autonomous inter-spheroid fusion as early as day 2, a deficit that persisted through the 4-day and 7-day checkpoints (Table 8).

Conversely, continuous storage at ambient temperature successfully preserved all critical quality attributes over the entire tested storage window. At days 2, 4, and 7, ambient-stored spheroids consistently maintained robust MTT viability, retained their full autonomous fusion potential, and preserved their internal GAG payload.

Quantitative biochemical tracking further defined the temporal limits of this ambient-temperature storage. While the total intra-spheroid GAG content remained remarkably stable (maintaining approximately 20 µg/spheroid) from day 0 out to day 14 (p > 0.05 across timepoints, via a repeated-measures ANOVA, n=3 biological replicates), a progressive accumulation of GAGs leaching into the surrounding storage medium was observed over time. GAG leakage into the medium became detectable by day 7 and essentially doubled by day 14 (p < 0.05, via a repeated-measures ANOVA, n=3 independent batches), identifying a clear 7-day kinetic boundary for optimal liquid storage to ensure the full retention of the biochemical payload within the construct prior to implantation.

The validation of a 7-day, ambient-temperature stability window in a minimal medium represents a massive logistical advantage for an investigational ATMP. This extended shelf-life entirely circumvents the need for complex, costly active cooling systems during hospital distribution. More importantly, it provides a highly practical temporal buffer necessary to complete rigorous batch-release quality control (QC) testing, such as endotoxin limits, sterility assurance, and rapid GAG quantification, and facilitates flexible surgical scheduling prior to patient administration in the operating room.

**Figure 4:**
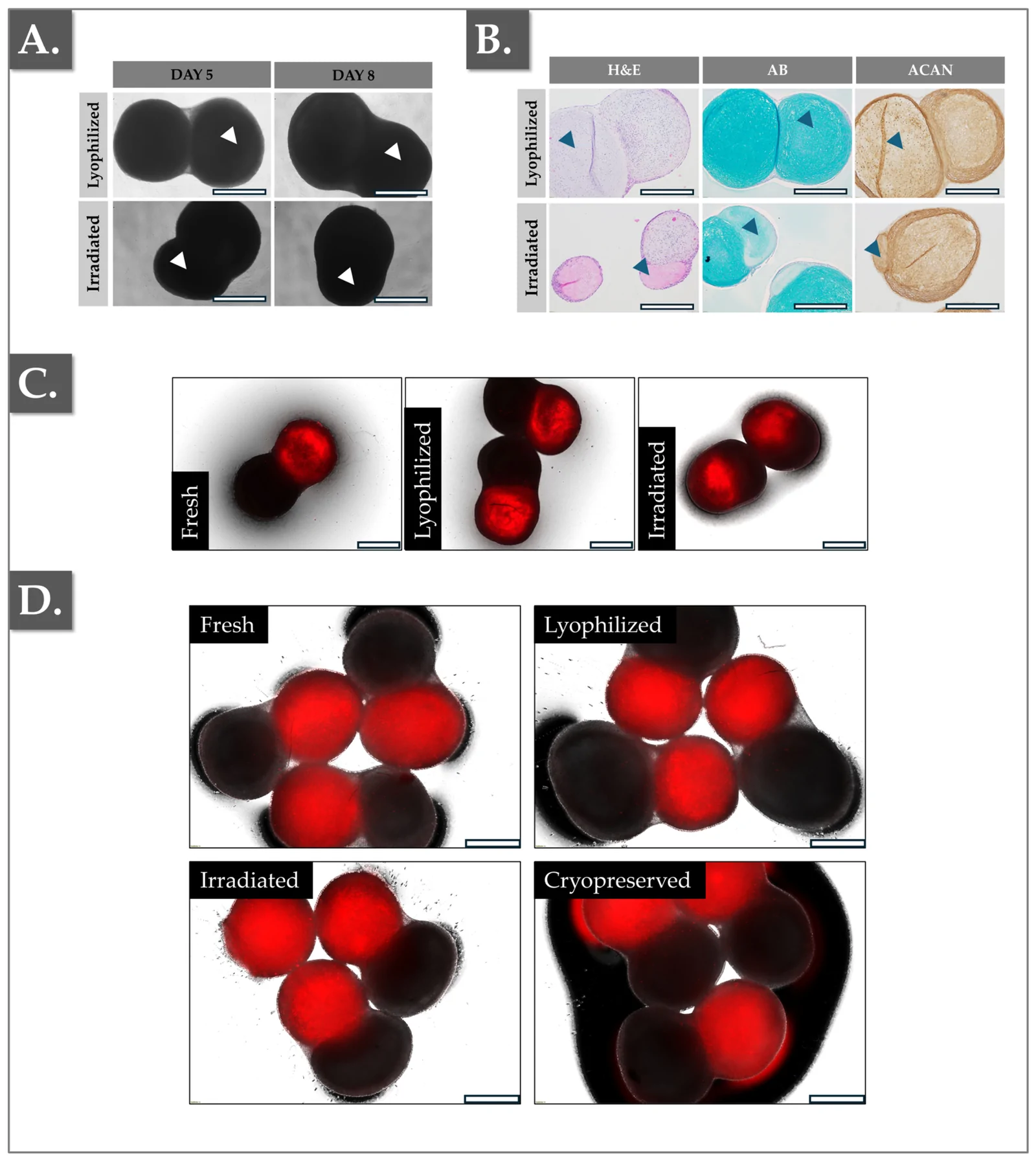
Heterologous structural merging and fusion potential of fresh spheroids with lyopreserved microtissues. (**A**) Macroscopic evaluation of autonomous tissue fusion between viable, fresh spheroids and non-viable, preserved constructs. Fresh spheroids were co-cultured with either lyophilized (top row) or lyophilized and gamma-irradiated (bottom row) spheroids. Brightfield imaging at day 5 and day 8 post-co-culture demonstrates successful structural merging. White arrowheads denote the processed (lyophilized/irradiated) spheroids. Notably, the macroscopic fusion process appears to occur more rapidly when utilizing terminally sterilized spheroids compared to lyophilization alone. (**B**) Histological characterization of the fused microtissue assemblies. Cross-sections were stained with Hematoxylin and Eosin (H&E), Alcian Blue (AB) for sulfated GAGs, and immunohistochemistry for aggrecan (ACAN). Dark blue arrowheads denote the processed (lyophilized/irradiated) spheroids. While lyophilized spheroids retained baseline matrix architecture (positive AB and ACAN staining), subsequent terminal sterilization severely degraded the structural integrity and ECM payload of the construct. (**C**,**D**) Fluorescent tracking of cellular integration utilizing CellBrite red membrane dye. Fresh, viable spheroids were fluorescently labeled (red) and placed in direct physical contact with unlabeled fresh, lyophilized, lyophilized and irradiated, or cryopreserved spheroids. Images acquired at day 5 post-fusion depict (**C**) dual-spheroid pairings and (**D**) multi-spheroid clusters. The fluorescent assays confirm that while the advanced preservation strategies (lyophilization and irradiation) eliminate intrinsic cellular viability, the resulting matrices successfully serve as integrative, bioactive scaffolds capable of heterologous fusion with viable cells. Scale bars = 500 μm. Representative macroscopic and fluorescent images were acquired from n=3 independent biological experiments to ensure reproducibility.

To explore the absolute limits of storage extension and evaluate the feasibility of high-dose gamma irradiation, the spheroids were subjected to lyophilization (freeze-drying). However, this extreme physical processing significantly compromised the autonomous regenerative capacity of the cellular formulation. Lyophilized spheroids lacked viable surface cells, as indicated by a negative MTT signal across *n* = 3 independent lyophilization runs, and subsequently lost the ability to autonomously fuse with one another. Interestingly, fresh viable spheroids retained the capacity to structurally fuse with the lyophilized constructs, suggesting that while the biological “engine” of the lyophilized 3D microtissue is disabled, the structure itself can still function as an integrative bioactive scaffold (Figure 4).

Notably, this heterologous fusion process appeared faster and more complete when the lyophilized spheroids had been irradiated at a dose of 31.5 kGy (reproducibly observed across *n* = 3 independent co-culture experiments; Figure 4A). Histological analyses further defined the structural toll of this advanced preservation strategy (Figure 4B). Lyophilized spheroids exhibited decreased nuclear staining (via H&E) compared to fresh controls, though they successfully retained positive Alcian Blue (AB) staining, indicating the initial preservation of sulfated GAGs. However, subsequent gamma irradiation of the lyophilized constructs severely degraded the ECM payload. Irradiation restricted observable nuclear staining and strongly reduced both AB and aggrecan histological signals, indicating a massive reduction in intact ECM (Figure 4B). These findings define the current physical boundaries of the 3D microtissue formulation: while short-term room-temperature liquid storage is highly viable and clinically advantageous, long-term preservation via lyophilization and terminal sterilization requires extensive formulation optimization to protect the complex, structurally vital ECM.

## 4. Discussion

Current clinical management options for cartilage lesions are fundamentally limited. Conservative treatments, such as physical therapy and analgesics, provide temporary symptomatic relief but fail to heal the underlying structural lesion [15]. While surgical interventions like microfracture (MFX) and ACI can offer structural repair, their well-documented clinical limitations, such as donor-site morbidity and 2D cellular dedifferentiation, necessitate more advanced approaches. Furthermore, while MFX remains a first-line therapy, it inherently lacks the precise microenvironmental cues required to drive true hyaline chondrogenesis, consistently resulting in the generation of mechanically inferior fibrocartilage, rich in type I collagen [6,7,11,40,57]. Indeed, recent Phase III randomized trials have demonstrated that implanting 3D chondrocyte spheroids yields superior clinical outcomes and better morphological repair quality than traditional MFX in long-term follow-ups [38]. To overcome these severe clinical limitations and biomanufacturing bottlenecks, this study engineered a highly organized, scaffold-free 3D spheroid formulation utilizing the characterized allogeneic FE002-Cart chondroprogenitor cell type, effectively bypassing the limitations of traditional scaffold-dependent platforms.

### 4.1. Microenvironmental Engineering: Defining Hypoxic and Glucocorticoid Thresholds

The phenotypic stability and ECM payload of the 3D microtissues are highly sensitive to manufacturing microenvironments, requiring precise control of CPPs to govern chondrocyte redifferentiation and matrix synthesis.

#### 4.1.1. The Active Role of Physiological Hypoxia

In their native articular cartilage environment, chondrocytes reside in a relatively hypoxic niche. This low-oxygen environment is not merely a passive characteristic, but an active, fundamental regulator of chondrocyte metabolism and function. This physiological response is mediated primarily by the transcription factor hypoxia-inducible factor-1 alpha (HIF-1α) [58]. HIF-1α plays a central role in regulating the expression of genes critical for chondrogenic differentiation, robust ECM synthesis, and cellular survival. Consequently, numerous studies have demonstrated that hypoxic conditions significantly enhance chondrocyte differentiation and promote the expression of key cartilage-specific genes, such as *COL2* and *ACAN* [34,43,44].

However, the exact optimal oxygen concentration for in vitro chondrogenesis remains debated in the literature, typically spanning a broad range from 1% to 10% O_2_ (e.g., 1% in Öztürk et al., 2.5% in Martinez et al., and 5% in Shi et al. or Martinez-Armenta et al.) [30,35,45,59]. This study expanded upon previous findings by demonstrating that the degree of hypoxia exerts a profound and direct influence on spheroid development, particularly concerning the deposition of GAGs and COL2 [60]. We established that strict physiological hypoxia at 2% O_2_ represents the optimal condition for FE002-Cart cells. Maintaining this specific 2% threshold maximized both GAG and COL2 synthesis, whereas progressively increasing oxygen levels (e.g., to 5% or 10%) negatively impacted cellular redifferentiation. This underscores the necessity of precise oxygen tension control to accurately mimic in vivo conditions, stabilize the mature chondrocytic phenotype, and achieve the desired tissue-engineered outcome [44]. Mechanistically, the profound HIF-1α stabilization observed at 2% O_2_ not only drives the transcription of structural matrix genes, but it also induces a fundamental shift in the cellular metabolic state. In the avascular core of a dense 3D spheroid, this HIF-1α-mediated transition toward anaerobic glycolysis is an adaptive necessity. It ensures that the encapsulated cellular payload can maintain the bioenergetic requirements necessary for robust, autonomous ECM assembly, while actively preventing central tissue necrosis within the nutrient-restricted microenvironment.

Importantly, our data highlight a necessary paradigm shift in 3D microtissue manufacturing: transient transcriptomic activity does not perfectly correlate with ultimate functional matrix deposition. Although 10% O_2_ generated the highest *ACAN* mRNA fold-induction, 2% O_2_ was designated as the optimal threshold because it maximized actual GAG and COL2 protein synthesis. Visually intense histological staining in stunted constructs (such as those at 10% O_2_) can artificially obscure overall production deficits. By prioritizing the absolute quantitative biochemical payload (total µg GAGs per spheroid) over relative mRNA expression, this manufacturing framework ensures the formulation of a mechanically robust and functionally superior engineered microtissue.

Beyond orchestrating structural matrix synthesis, the activation of the fundamental HIF-1 signaling pathway unmasked distinct secretome dynamics in our 2D cellular active substance characterization. Specifically, a deep hypoxic microenvironment (2% O_2_) actively triggered the upregulation of potent angiogenic factors, including a progressive elevation of VEGF-A and a highly specific > 3-fold upregulation of VEGF-C relative to normoxic controls. Because native articular cartilage is strictly avascular, aneural, and relies entirely on passive nutrient diffusion, the production of angiogenic secretome components introduces a critical functional paradox in cartilage tissue engineering. However, existing literature indicates that transient VEGF expression is a natural byproduct of robust HIF-1α stabilization during the initial expansion and early differentiation phases of chondroprogenitors [58].

Crucially, while freely suspended cells might risk promoting vascular invasion or endochondral ossification via localized VEGF secretion, our finalized scaffold-free 3D microtissue formulation delivers cells embedded within a dense, pre-formed 3D ECM network. This highly cohesive 3D physical architecture restricts cellular payload dispersion. Furthermore, the literature suggests that mature, localized ECM payloads, dense with synthesized aggrecan and specialized GAGs, act as a functional physical and biochemical barrier that suppresses endothelial cell infiltration and actively blocks neo-vascularization in vivo [61]. Consequently, the temporary upregulation of VEGF components prior to 3D tissue assembly reflects an active, upstream signaling cascade that does not compromise the long-term structural or avascular phenotypic stability of the mature spheroid implant.

#### 4.1.2. Defining the Glucocorticoid Threshold

Concurrently, biochemical signaling, specifically via synthetic glucocorticoids, acts as a master threshold for 3D tissue assembly. Dexamethasone functions as a potent modulator by interacting with intracellular glucocorticoid receptors, triggering a cascade of downstream events that ultimately influence the expression of cartilage-specific genes and ECM deposition [62]. The concentration of dexamethasone within the differentiation medium must be strictly regulated, as both insufficient and excessive levels disrupt the delicate balance of chondrogenic signaling, leading to suboptimal tissue formation [46].

While high concentrations (e.g., 100 nM) are commonly utilized in standard chondrogenic media, the literature indicates these supraphysiological doses can paradoxically inhibit cartilage formation and disrupt intricate regulatory networks, particularly in mesenchymal stem cells (MSCs) as noted by Chijimatsu et al. [46]. Conversely, optimization studies suggest a highly effective concentration range between 10 and 50 nM [47]. Our data confirmed that a 100 nM dose is actively detrimental to optimal FE002-Cart spheroid formation, mirroring observations in 3D mesenchymal pellets where high-dose glucocorticoids induced central tissue necrosis and fibrotic cores and corroborating similar deleterious effects on COL2 deposition previously observed with the related FE002-Disc cell type [46,60]. Fine-tuning the dexamethasone concentration to exactly 10 nM was a necessity for maximizing robust GAG accumulation and ensuring the appropriate, homogeneous deposition of collagen type II, while minimizing potential off-target effects.

This strict requirement to lower and precisely control the glucocorticoid threshold to exactly 10 nM carries critical functional implications for preventing terminal hypertrophic degradation. Synthetic glucocorticoids like dexamethasone are notoriously potent dual-regulators in skeletal tissue engineering; while they are essential for initiating early chondrogenic signaling networks, high doses are heavily linked in the literature to pushing cells toward late-stage endochondral ossification [63,64]. This hypertrophic path is characterized by a dramatic upregulation of type X collagen (COL10) and matrix metalloproteinase 13 (MMP-13), key degradative markers that must be actively suppressed by optimized microenvironments, which destabilizes the tissue, induces cell death, and ultimately leads to calcification of the newly synthesized matrix [24,44,45].

Our comparative results with primary adult chondrocytes highlight this vulnerability, as adult donor cell types frequently displayed an unwanted upregulation of *COL10A1* under unoptimized or erratic conditions, indicating a strong propensity for terminal phenotypic drift. By fine-tuning and limiting the biochemical exposure to a strict 10 nM window, we successfully balanced these competing signaling pathways. Literature indicates that minimizing synthetic glucocorticoid levels to a physiological range maintains downstream *Sox9* transcript activity, which preserves the functional, hyaline-like matrix backbone, while actively dampening the hyper-activation of downstream *Runx2* transcription cascades that drive hypertrophy [65,66,67]. Consequently, this stringent boundary does not merely optimize matrix accumulation; it functions as a critical biochemical checkpoint that suppresses terminal hypertrophy and supports the phenotypic stability of the allogeneic FE002-Cart formulation during the in vitro maturation phase.

From a temporal perspective, the stringent combination of 2% O_2_ and 10 nM dexamethasone plays distinct mechanistic roles during different phases of microtissue development. During the initial manufacturing phase, these highly defined microenvironmental cues act as potent triggers for early chondrogenic commitment, rapidly initiating the *Sox9* signaling cascade and driving the exponential synthesis of COL2 and ACAN. However, their continuous presence throughout the 3-week culture period is equally important for late-stage tissue maturation. Rather than solely pushing further differentiation, these parameters most likely function as a sustained biochemical brake during the maturation phase. Therein, they are thought to actively stabilize the mature chondrocyte phenotype and prevent the newly formed microtissues from progressing into endochondral ossification, thereby ensuring the structural integrity of the final engineered microtissue.

### 4.2. Overcoming Translational Bottlenecks: The Transition to GMP-Compatible Excipients

Translating a cell-based therapy from the laboratory bench to clinical-scale manufacturing requires the systematic substitution of research-grade reagents with GMP- or pharmaceutical-grade equivalents. GMP-grade reagents are produced under stringent quality control measures to ensure safety, efficacy, and reproducibility, meeting the rigorous regulatory requirements for clinical applications. This transition, however, represents a critical scientific hurdle, as subtle variations in pharmaceutical excipients, stabilizers, and proprietary formulations can inadvertently alter complex cellular signaling pathways [68].

Our systematic qualification of raw materials aimed to transition the highly optimized research differentiation medium into a fully defined, clinical-grade formulation. Initial formulation optimization focused on the ITS supplement, vitamin C, and dexamethasone sources. High variability was uncovered concerning the dexamethasone constituent of the chondrogenic medium. These findings emphasize that rigorous raw-material qualification and the systematic biological evaluation of each individual component are mandatory operational standards in ATMP manufacturing [69]. Careful selection of GMP-grade components ensures that clinical excipients do not compromise the phenotypic identity, matrix quality, structural integrity, efficacy, or safety profile of the final therapeutic product.

#### 4.2.1. Securing the Biomanufacturing Supply Chain

Beyond the initial validation of a single clinical-grade formulation, establishing a truly robust biomanufacturing process mandates the proactive identification and parallel qualification of multiple redundant reagents for each critical manufacturing step. Relying exclusively on a single commercial supplier for specialized GMP-grade excipients introduces a severe translational vulnerability. Unforeseen supply chain disruptions, unannounced proprietary formulation changes, or the sudden commercial discontinuation of a critical reagent can abruptly halt clinical manufacturing and compromise patient care. By successfully qualifying multiple functional equivalents, such as validating various clinical-grade insulin formulations in parallel, manufacturers can preemptively secure the supply chain, mitigate logistical risks, and guarantee continuous production.

#### 4.2.2. Methodology for Reagent Comparability

When qualifying these redundant components or substituting a discontinued reagent, the analytical methodology must rigorously adhere to the principles outlined in the International Council for Harmonisation (ICH) Q5E guidelines (Comparability of Biotechnological/Biological Products Subject to Changes in their Manufacturing Process). The introduction of any alternative raw material necessitates a comprehensive comparability exercise to definitively prove that the new reagent does not adversely impact the quality, safety, or efficacy of the cellular active substance [70]. This requires parallel, head-to-head manufacturing runs assessing highly specific quality attributes, such as cellular viability and morphology (ensuring the structural consolidation of the 3D microtissues remains unaffected), biochemical payload (quantifying specific matrix deposition, including total GAGs and COL2 accumulation), and transcriptional profiling (verifying that chondrogenic gene induction remains stable and that terminal hypertrophic markers are not inadvertently upregulated). Applying this strict comparability framework ensures the therapeutic product remains highly consistent and functionally reliable, regardless of which specifically qualified raw material is utilized during the manufacturing run.

### 4.3. The Paradigm Shift from 2D Cell Suspensions to 3D Scaffold-Free Microtissues

Securing a robust, GMP-compatible biochemical supply chain ensures the safe manufacturing of the active substance; however, the ultimate therapeutic potential of this engineered formulation relies heavily on its physical delivery format. The strategic decision to formulate therapeutic cells as 3D microtissues rather than standard 2D injectable cell suspensions represents a pivotal consideration in the design of regenerative therapies for articular cartilage. When isolated and cultured in a conventional 2D environment, chondrocytes rapidly undergo dedifferentiation [10,11]. This process is characterized by a phenotypic shift away from their specialized state and a diminished expression of crucial markers, most notably the structural backbone protein, collagen type II (COL2) [1,10,12,14]. This dedifferentiation is notoriously difficult to reverse, as even under physiological hypoxia, chondrocytes on 2D surfaces fail to re-differentiate and instead exhibit detrimental upregulation of collagen type I [35,59].

Conversely, our 3D formulation efficiently re-establishes the complex spatial architecture required to restore a native-like chondrocyte identity, a restorative capacity previously observed in autologous spheroid models [10,12,29,30,45,65]. Mechanistically, encapsulating chondrocytes in a 3D microenvironment acts synergistically with hypoxia to suppress negative regulatory pathways, such as RhoA and canonical Wnt/β-catenin signaling, thereby enabling robust expression of collagen type II and aggrecan [59]. The spatial organization afforded by 3D systems also allows for the establishment of cell polarity, the formation of critical intercellular junctions, and the development of dense paracrine signaling networks [71].

Furthermore, maintaining these biomechanical and structural properties in 3D is critical for the construct to successfully withstand the compressive forces native to the joint [9]. These conditions robustly promote the re-expression of key phenotypic markers, including *COL2* for tensile strength and *ACAN* for compressive resilience through water retention [72]. Furthermore, the transition to 3D culture induces profound alterations in the cellular proteome, resulting in a protein expression profile that more faithfully recapitulates the complex biochemical machinery of native tissue homeostasis [73,74]. Of note, in the present study, spheroids with high maturity were less prone to homologous fusion. This is likely attributable to the progressive accumulation of dense ECM as the spheroids mature, which acts as a physical barrier, restricting cellular mobility and reducing the number of cells available to migrate and bridge adjacent spheroids, a phenomenon recently quantified in other modular systems, where increased cortical actin and ECM density actively restricted inter-spheroid fusion kinetics [17,19].

Beyond restoring cellular identity, the re-differentiation of cells within a 3D construct delivers both a viable cell population and a nascent cartilage-like ECM directly to the defect site in a highly cohesive format that can withstand the physical shear forces of minimally invasive clinical delivery, adhering to the defect naturally to facilitate scaffold-free arthroscopic implantation [31,37,39,53,75]. This pre-formed matrix provides immediate structural support and acts as a localized reservoir for concentrated, bioactive signaling molecules that can instruct surrounding host cells and guide endogenous repair [2,50]. Crucially, this 3D ECM functions as a physical shield. Following implantation, the engineered matrix must withstand the previously described catabolic joint milieu, which is rich in degradative enzymes and mechanical stresses [43]. While freely suspended 2D cells often suffer from rapid clearance, massive viability loss, and dispersion from the defect site due to an inability to withstand uncontrolled in vivo environments, cells embedded within a 3D matrix are afforded substantial physical shielding [22,50]. This retained endogenous ECM vastly improves cell survival and stable integration into the host tissue compared to traditional 2D monolayers [52].

Our functional assays confirmed that the FE002-Cart 3D formulation exhibits remarkable phenotypic resilience. When directly exposed to 100% OA synovial fluid, a severely catabolic environment, the spheroids maintained a high degree of metabolic viability, proving the inherent protective capacity of the 3D structure. Furthermore, a nuanced evaluation of our in vitro stress assays unmasked a critical mechanistic distinction regarding how the 3D ECM protects the encapsulated cellular payload from joint-specific pathologies. Our in vitro stress assays revealed that while the 3D matrix is metabolically vulnerable to direct enzymatic and catabolic cytokine signaling, it effectively buffers the severe shifts in microenvironmental pH caused by lactic acid [76,77].

In the context of an osteoarthritic joint, pro-inflammatory cytokines such as TNF-α, IL-1β, and IL-6 actively upregulate downstream MMPs and aggrecanases, which directly target and cleave structural proteoglycans [21,49,78]. The fact that our engineered spheroids successfully maintained robust overall metabolic viability and preserved their primary tissue architecture under these severe cytokine challenges highlights their structural resilience compared to traditional, unprotected 2D-grown cell therapies. This phenomenon is consistent with recent findings that dense 3D chondrospheres can withstand highly acidic and inflamed degenerative microenvironments without losing viability [53,54]. However, the observed GAG loss under direct inflammatory stress indicates that while 3D structural pre-differentiation provides a vital protective boundary at the defect site, achieving optimal clinical outcomes in highly active osteoarthritic environments may necessitate pairing the ATMP implantation with concurrent anti-inflammatory therapies to fully safeguard the newly delivered matrix payload from catabolic degradation [51].

Furthermore, these spheroids retained the capacity for active cellular egress, functioning as modular tissue units demonstrating the in vitro capacity to structurally merge with adjacent microtissues, aligning with foundational observations that scaffold-free chondrocyte spheroids can rapidly form focal adhesions and integrate into native osteoarthritic cartilage explants [10,18,50]. This also aligns with recent literature demonstrating the robust autonomous fusion kinetics of scaffold-free chondrospheres upon injection [53,54]. Interestingly, cellular migration kinetics appear to be inversely proportional to the construct’s maturation stage, suggesting that tuning the maturation timeline prior to implantation could serve as a strategy to precisely optimize regenerative potential. This concept was recently corroborated by retrospective clinical data showing that excessively mature spheroids significantly increase patient non-responder rates [79].

Accumulating preclinical and clinical evidence strongly underscores the greater therapeutic potency of 3D cell delivery over 2D cell suspensions. In vivo animal models, such as rat osteochondral defect studies, have consistently demonstrated that implanting cells within a 3D matrix yields superior, more organized cartilage repair compared to the direct injection of 2D-expanded cells [29]. Specifically, in vivo models comparing monodispersed cell suspensions versus pre-formed 3D spheroids demonstrate that the spheroid configuration significantly enhances cell proliferation, upregulates chondrogenic genes (*COL2A1*, *SOX9*), inhibits hypertrophic markers (*COL10A1*), and forms biomimetic lacunae that closely resemble natural cartilage [32]. This superior efficacy is increasingly supported by clinical trial data. For instance, a prospective trial comparing the implantation of mature tissue-engineered cartilage (N-TEC, a 3D product) against undifferentiated nasal chondrocytes (N-CAM, a less structured 2D-like cell product) reported significantly higher patient-reported functional scores (KOOS) and vastly superior structural and compositional repair (assessed via 3D-MOCART imaging) for the fully organized 3D formulation [80].

### 4.4. Benchmarking In Vitro Biosynthesis: Spheroids vs. Matrix-Assisted Technologies

To validate the clinical utility and scientific superiority of the scaffold-free spheroid strategy, we systematically benchmarked its biosynthetic efficiency data against standard reported matrix-induced autologous chondrocyte implantation (MACI) analogs. Previous work demonstrated the suitability of FE002-Cart cells for seeding on a Chondro-Gide collagen membrane to form implantable constructs, a matrix platform clinically used by Mumme et al. for autologous nasal chondrocyte grafting [24,28]. However, our current findings reveal that these cells exhibit greater regenerative potential when formulated as 3D spheroids, demonstrating enhanced ECM deposition and functional metrics compared to the Chondro-Gide combination product.

A critical advantage of the spheroid system is its capacity to promote robust collagen type II (COL2) induction and ensure a highly uniform distribution of this essential matrix component. COL2 is the principal fibrillar collagen of articular cartilage, directly providing the tissue with its characteristic tensile strength and structural integrity [81]. In sharp contrast to the heterogeneous and uneven deposition frequently observed on scaffold-based systems like Chondro-Gide, the 3D spheroids displayed strong, homogeneous COL2 deposition throughout their structure. This structural uniformity suggests the formation of a more physiologically relevant tissue and is highly likely to translate into improved biomechanical properties and ensure the long-term functional stability of the engineered cartilage.

Furthermore, the biosynthetic efficiency of the spheroid platform is dramatically higher when normalized to the cellular starting material. We observed a massive increase in the deposition of GAGs, such as aggrecan and hyaluronan, which are highly negatively charged polysaccharides crucial for attracting and retaining water within the tissue. This water retention is the fundamental mechanism providing cartilage with its compressive resilience against dynamic joint loads [82].

Quantitatively, seeding 1 million FE002-Cart cells onto a Chondro-Gide membrane typically yields approximately 50 µg of GAGs [28]. Thus, when normalized per cell, the membrane-based protocol yields approximately 0.05 ng of GAGs per cell. In stark contrast, an individual spheroid containing 30,000 cells produces roughly 15 µg of GAGs, equating to 0.5 ng of GAGs per cell. This represents a tenfold increase in GAG production efficiency per cell, indicating a substantially higher capacity for GAG accumulation, which is a known biological prerequisite for water retention in engineered cartilage formulations. However, the historical Chondro-Gide control data were obtained with constructs maintained under a shorter induction timeline (16±4 days) compared to the 3-week maturation period utilized for the 3D spheroids [28]. Furthermore, the matrix-assisted protocols utilized human platelet lysate (HPL) and a higher concentration of dexamethasone ($10^{-7}$ M/100 nM), whereas the optimized spheroid manufacturing protocol utilized a defined medium containing 10 nM dexamethasone and TGF-β3 [28]. Overall, these divergent timelines and biochemical microenvironments represent methodological limitations in direct side-by-side benchmarking, indicating that differences in baseline matrix yield may be partly influenced by variations in media composition and culture duration rather than geometry alone.

Notwithstanding, this enhancement in cell-normalized biochemical output unmasks a fundamental mechanobiological advantage inherent to scaffold-free 3D microtissue assembly. When therapeutic cells are seeded onto standard porous biomaterial membranes like Chondro-Gide, they are initially physically isolated from one another, separated by large artificial gaps within the exogenous matrix. This sparse spatial distribution forces the cells to expend significant metabolic energy adhering to and navigating the foreign structural network before establishing a homeostatic baseline, which frequently results in poor phenotype maintenance and histological structures that fail to resemble native cartilage [32].

In sharp contrast, the scaffold-free aggregation of 30,000 cells into an intimate, dense 3D spheroid structure completely eliminates this artificial interface. By placing single cells into immediate, unhindered physical proximity, the spheroid configuration perfectly recapitulates the embryonic precartilage condensation phase that occurs during native limb bud development [18,19,33,34]. This high-density cell-to-cell interaction triggers immediate, cadherin-mediated intercellular signaling and forms rich paracrine networks that rapidly upregulate downstream chondrogenic master regulators. Consequently, instead of losing metabolic efficiency to scaffold attachment, the cellular payload is immediately directed toward autonomous ECM synthesis, resulting in the massive, homogeneous accumulation of COL2 and GAGs observed in our results. This biomimetic shift from a matrix-dependent system to an autonomous, condensed cell community explains the remarkable biosynthetic efficiency of the spheroid platform, demonstrating a robust in vitro functional payload and substantially reducing the risks of suboptimal degradation kinetics or immune responses associated with artificial scaffold materials [9,15].

### 4.5. Comparison with Current Commercial and Clinical Cartilage Spheroids

Contextualizing the studied FE002-Cart engineered microtissue against existing clinical-stage spheroid products underscores its therapeutic potential and unique profile within the landscape of cartilage repair strategies. A critical aspect of this comparison centers on the GAG content of the engineered tissue, as GAGs contribute significantly to the compressive resilience and load-bearing capacity of articular cartilage.

#### 4.5.1. Existing Spheroid-Based Therapies

Spherox, a leading autologous scaffold-free 3D spheroid product approved by the EMA and reviewed for efficacy against MFX in the treatment of articular cartilage defects, utilizes a 14-day differentiation period to produce spheroids of approximately 200,000 cells containing 0.5 to 2.5 µg of GAGs [11,23,38]. The intervention has demonstrated long-term safety across various clinical dosing regimens and serves as a highly effective midterm treatment for large cartilage defects with remarkably low failure rates [36,37]. Crucially, research on Spherox has identified a positive correlation between ACAN content prior to implantation and the potential for successful in vitro defect filling, underscoring the absolute importance of robust proteoglycan synthesis for regenerative capacity [42]. Furthermore, clinical investigations evaluating Spherox manufacturing parameters have revealed that excessively prolonged 2D and 3D culture times correlate negatively with the Knee injury and Osteoarthritis Outcome Score (KOOS) [79]. This highlights the risk of over-differentiation or cellular senescence, thereby necessitating strict donor cell qualification to ensure cells possess optimal proliferative and differentiation potential.

Cartibeads, currently undergoing clinical trials, implements a specific 2D culture protocol allowing extensive cell amplification and banking while highly effectively preserving chondrogenic potential [83]. This product generates spheroids of roughly 200,000 cells with an elevated GAG content of approximately 40 µg after 2 weeks of 3D culture [13]. To address patient-to-patient biological variability in allogeneic applications, Cartibeads trials incorporate a rigorous preselection step to identify specific donors with high chondrogenic potential, aiming to improve the consistency and predictability of treatment outcomes. Encouragingly, these microtissues have recently demonstrated robust in vivo efficacy, achieving stable integration and native-like structural reorganization in large animal models [75].

Another pipeline therapy, CCP-ACI (NCT03517046), utilizes autologous costal chondrocytes, derived from rib cartilage to potentially offer cell availability and proliferative advantages over articular sources. This process yields spheroids of 100,000 cells containing roughly 14 µg of GAGs after a 10-day differentiation period [39]. In a prospective randomized trial comparing this platform directly to microfracture for defects spanning 2–10 cm^2^, the spheroid formulation met its primary clinical endpoints, exhibiting no treatment-related serious adverse events while progressively driving hyaline-like matrix filling [40,84]. Preclinical tumorigenicity assessments in mice and minipigs, alongside early clinical trials evaluating varying therapeutic doses over multi-year follow-ups, have demonstrated the general safety and efficacy of these spheroid-based products in alleviating pain, improving joint function, and promoting regeneration [31,36].

#### 4.5.2. The FE002-Cart 3D Microtissue Advantage

Compared to these existing products, the investigational FE002-Cart engineered microtissue utilizes a proportionally lower cellular starting material load per microtissue, which could potentially simplify manufacturing processes and reduce cell culture costs. Despite the lower initial cell density, these spheroids deliver a remarkably dense, hyaline-like matrix payload, exhibiting highly robust GAG accumulation. This indicates a proportionally higher capacity for GAG production per cell.

While the FE002-Cart differentiation timeline is generally longer than that employed for some other spheroid therapies, this extended period actively contributes to the increased GAG accumulation, allowing cells sufficient time to synthesize and deposit complex matrix components. Based on the correlations established with Spherox in ex vivo human defect models, the robust ACAN and GAG content strongly suggests a high capacity for in vivo cartilage regeneration and defect filling [42]. Furthermore, packaging these standardized allogeneic cells into an autonomous, scaffold-free 3D microtissue aligns perfectly with the field’s rapid evolution toward ‘fourth-generation’ cartilage therapies, circumventing the regulatory and surgical complexities associated with older exogenous matrices [25,27,83].

#### 4.5.3. Circumventing the Autologous Bottleneck: The Imperative for Allogeneic Consistency

A fundamental limitation of autologous, patient-derived cartilage cell therapies is the inherent biological unpredictability of the starting material [26,79]. Our comparative analysis illustrated this clinical bottleneck: while the allogeneic FE002-Cart formulation consistently yielded cohesive microtissues with uniform structural and biochemical profiles, primary adult chondrocytes exhibited profound inter-donor heterogeneity. This variability manifested in extreme functional fluctuations, with mature adult cohorts producing GAG yields that ranged erratically from a severely depressed 6.5 µg to 40 µg per spheroid under identical, optimized manufacturing parameters.

More critically, this morphological inconsistency was mirrored at the transcriptional level. Even under strictly controlled hypoxic conditions (2% O_2_), multiple adult donor cell types displayed an alarming propensity for phenotypic drift, marked by the spontaneous upregulation of fibrocartilage (*COL1A1*) and terminal hypertrophic (*COL10A1*) markers. This phenotypic instability in adult autologous cells is frequently documented in the literature and is driven by the compounded effects of age-related cellular senescence, cumulative mechanical stress, and epigenetic alterations stemming from pre-existing joint comorbidities [21,49].

Such stark biological unpredictability poses a severe regulatory and clinical risk, as it precludes the ability to guarantee a uniform therapeutic payload or a stable clinical outcome from batch to batch. In contrast, the highly characterized, banked FE002-Cart cell type actively resisted this phenotypic degradation, delivering stable, targeted hyaline cartilage induction across all manufacturing replicates. Consequently, utilizing this allogeneic source effectively circumvents the logistical necessity for repeated rigorous donor pre-screening, and supports the lot-to-lot consistency required for standardized biomanufacturing [26].

#### 4.5.4. Preclinical Biosafety and Forward Outlook

From a safety perspective, the 3D manufacturing process did not induce changes in telomerase activity, a crucial marker of cellular immortality and a potential indicator of tumorigenesis, after the 3D culture phase. Additionally, previous in vivo animal models using 2D FE002-Cart cells did not reveal any signs of tumorigenicity [85]. This preclinical safety profile aligns with the extensive safety data available for other spheroid therapies (e.g., Spherox, CARTISTEM, TissueGene-C, Cartibeads) [13,20,24,36,41]. However, rigorous in vivo studies with long-term follow-ups and well-designed clinical trials remain essential to definitively establish the clinical efficacy, including targeted pain relief and functional improvement, and the long-term safety of this novel 3D microtissue. Indeed, recent health technology assessments of existing autologous spheroid therapies highlight that the absence of long-term (e.g., > 5 years) efficacy data remains one of the most significant regulatory and commercial barriers to widespread clinical adoption [23].

### 4.6. Resolving Supply Chain Logistics and Defining Release Criteria

Establishing the biological superiority and inherent protective capacity of the 3D engineered microtissue fulfills the clinical requirements for cartilage repair. However, translating this prototype into a clinical reality requires resolving fundamental supply chain vulnerabilities. While conventional liquid storage protocols for standard 2D-expanded cellular suspensions heavily rely on continuous refrigeration (4 °C) to slow baseline metabolism and prevent rapid nutrient exhaustion, our data revealed that this strict cold-chain approach is actively detrimental to our dense, 3D scaffold-free microtissues. The severe impairment of autonomous inter-spheroid fusion observed as early as day 2 under 4 °C storage highlights a critical mechanobiological vulnerability. In highly organized 3D cellular constructs, structural maintenance and tissue integration rely on continuous, active cytoskeletal remodeling, cadherin-mediated cell sorting, and the constant recycling of cell-surface adhesion proteins. Dropping the ambient temperature to 4 °C effectively paralyzes this vital migratory and metabolic machinery [86,87].

Conversely, continuous storage at ambient temperature in a minimal basal medium successfully balances these biological demands. By maintaining the spheroids at ambient temperature, the cells sustain a low-level baseline metabolism that is robust enough to preserve the cytoskeletal dynamics and integrin expression required for autonomous host tissue integration, yet tempered enough to prevent the premature exhaustion of the limited nutrient supply [88]. This indicates that the logistical viability of 3D cellular therapies is governed by fundamentally different metabolic thresholds than single-cell suspensions, making ambient temperature transport not only a logistical convenience but a strict biological necessity for preserving baseline biological properties.

For rapid in-process monitoring, we identified robust QC parameters that perfectly align with clinical manufacturing constraints. Macroscopic monitoring of spheroid growth serves as a simple, non-invasive early warning system, as we observed a strong and consistent correlation between macroscopic spheroid size and GAG content. For a more direct and quantitative assessment, a 24 h biochemical GAG assay provides a reliable measure of matrix synthesis, making it a highly practical option for routine QC testing and a potential surrogate for final product release. This strategy directly aligns with established regulatory frameworks where pre-implantation matrix metrics are validated as predictive potency assays for structural cartilage repair [20,42].

To transition these inline analytical metrics from laboratory observations to validated pharmaceutical release criteria, they must be strictly mapped against established regulatory frameworks. The 7-day ambient temporal buffer successfully decouples manufacturing completion from surgical scheduling, providing the mandatory operational window to execute compendial safety testing. Specifically, safety release protocols must align with the European Pharmacopoeia (Ph. Eur.) and United States Pharmacopeia (USP) guidelines for cellular therapies, substituting traditional long-term microbiological cultures with rapid, validated alternatives. For instance, sterility assurance should be conducted in compliance with Ph. Eur. Chapter 2.6.27 (Microbiological Examination of Cell-Based Preparations) or via automated nucleic acid amplification techniques (NAT) under Ph. Eur. Chapter 2.6.21 to detect mycoplasma and slow-growing contaminants within the restricted shelf-life window. Concurrently, pyrogenicity and endotoxin thresholds must be quantified using the limulus amebocyte lysate (LAL) framework according to Ph. Eur. Chapter 2.6.14 (Bacterial Endotoxins)/USP 85, ensuring the active substance remains well below safety limits before hospital dispatch. By cross-referencing these precise pharmacopeial chapters, the 3D spheroid manufacturing pipeline establishes a legally compliant, reproducible path toward commercial batch release, satisfying a critical prerequisite for advanced therapy clinical translation.

Establishing a direct, linear correlation between macroscopic spheroid size and absolute internal GAG content represents a substantial advancement for inline analytical bioprocessing. In large-scale clinical manufacturing, routine batch release is frequently bottlenecked by the destructive, labor-intensive, and time-consuming nature of traditional biochemical assays like the DMMB protocol [14]. Utilizing non-invasive optical profiling, specifically measuring the Feret diameter and construct cross-sections, as an indicator of structural potency provides a rapid, non-destructive, and real-time analytical alternative.

While regulatory bodies might argue that macroscopic volumetric growth could be falsely driven by cellular swelling, loose cell aggregation, or internal central necrosis, our paired histological and biochemical data strongly suggest otherwise. Spheroids cultivated under optimal microenvironmental parameters demonstrated that increased physical cross-sections directly mirror true, homogeneous ECM accumulation and localized proteoglycan synthesis rather than artifactual fluid retention. This relies on validated principles in ATMP bioprocessing literature, where automated morphological imaging is increasingly recognized as a reliable, high-throughput tool to monitor tissue maturation. By integrating automated, non-invasive optical tracking into the manufacturing line, producers can rapidly establish product consistency and safely estimate the in vitro biochemical payload of individual batches without sacrificing valuable living tissue prior to surgical implantation [35].

While RT-PCR provides essential, detailed molecular profiling of chondrogenic gene expression (e.g., *COL2* and *ACAN*), and histological analysis reveals the precise spatial distribution of ECM components, their extended processing times and labor-intensive nature render them unsuitable for routine, rapid batch release. Nevertheless, they remain indispensable tools for in-process characterization and structural confirmation during process development, as rigorous validation of specific phenotypic markers (e.g., *COL2*, *ACAN*, *SOX9*) via these methods is universally recognized as critical for ensuring the safety and quality of engineered cartilage therapies [11].

To explore the absolute limits of shelf-life extension, we evaluated the feasibility of lyophilization (freeze-drying), which offers the prospect of storing therapies for months or years, facilitating massive batch production, extensive validation, and potentially high-dose gamma irradiation [22]. However, this extreme physical processing severely compromised the autonomous regenerative capacity of the cellular formulation. This dual capability of the preserved microtissues to achieve heterologous integration highlights an important paradigm shift in advanced shelf-life extension strategies. While the physical shock of freeze-drying and the high energy of gamma irradiation eliminate the biological “engine” of the formulation by destroying surface cell viability, they simultaneously transition the living 3D microtissue into a highly organized, off-the-shelf bioactive matrix. In the field of bone and cartilage reconstruction, traditional acellular scaffolds often fail because they lack appropriate architectural density or native-like extracellular signaling [89]. Our finding that fresh, viable spheroids can rapidly adhere to, remodel, and structurally merge with both lyophilized and gamma-irradiated constructs indicates that the synthesized matrix retains crucial biochemical cues, such as localized aggrecan and structural collagen frameworks, even after losing its cellular activity.

Interestingly, the macroscopically accelerated fusion kinetics observed when pairing fresh cells with terminally sterilized spheroids compared to lyophilization alone suggests that irradiation may induce subtle structural modifications. Gamma irradiation at standard sterilization doses (30 ± 5 kGy) likely introduces micro-fractionations or alters the cross-linking density of the dense ECM [90]. While this high-energy processing takes a clear structural toll by degrading native proteoglycan signals, the resulting increase in matrix porosity and the liberation of latent binding sites may paradoxically lower the physical resistance to cellular infiltration, allowing incoming viable cells to migrate and consolidate the boundary more rapidly. This dual behavior unmasks a unique translation opportunity, as even when a batch fails to meet the strict viability release criteria required for a living cytotherapy, the preserved constructs do not lose their therapeutic utility; instead, they could be repurposed as high-density, terminally irradiated cartilage-like matrices capable of autonomous in vitro structural merging. Therein, lyophilized or sterilized spheroid could potentially be used as “fillers” alongside a number of freshly cultured and metabolically viable spheroids, in order to rationalize both the manufacturing and clinical workflows, although this remains to be studied in ex vivo and in vivo models.

### 4.7. Limitations and Future Clinical Perspectives

Despite the substantial biological advantages of 3D microtissue biomanufacturing over traditional injectable 2D cell suspensions, the transition to this platform introduces specific economic and operational hurdles [27]. The overall production time for 3D constructs is inherently protracted. It necessitates an initial 2D expansion phase to amplify cell numbers, followed by a rigorous 2- to 3-week 3D differentiation phase to promote robust ECM formation. This extended timeline contributes to vastly increased manufacturing costs and limits the immediate, on-demand availability of the therapy for patients requiring urgent intervention. Furthermore, the process relies on specialized infrastructure, specifically incubators capable of precisely controlling and maintaining low-oxygen (hypoxic) conditions. This equipment is not universally available in standard cell culture facilities, adding significant capital expense and complexity to the production process.

The current production of 3D constructs also demands frequent, manual, and labor-intensive medium changes (e.g., three times weekly), which directly translates to increased labor costs and a higher consumption of expensive pharmaceutical-grade reagents. Future manufacturing iterations must focus on process intensification. For instance, investigating whether a reduced medium change frequency (e.g., twice weekly) is sufficient to maintain cell viability and matrix production without compromising product quality could significantly reduce overhead. Additionally, the manual nature of spheroid harvesting and medium exchange inherently increases the risk of microbial contamination and limits overall throughput. The implementation of closed-system automation for these steps is a critical necessity to improve efficiency, reduce labor costs, and guarantee product sterility [26]. Furthermore, while short-term liquid storage was validated, continued investigation into long-term preservation methods, such as optimized lyophilization or cryopreservation, remains essential to fully realize a widely distributable, off-the-shelf product. Current literature indicates that while the cryopreservation of spheroids for scaffold-free assembly is feasible, it introduces significant translational hurdles, including diminished post-thaw cellular viability and reduced mechanical tensile strength in the resulting tissue constructs [55]. Other reports show that standard cryopreservation induces severe cytoskeletal damage in spheroids, necessitating the development of advanced macromolecular cryoprotectants to successfully rescue 3D tissue viability [56].

On a technical note, while lyophilization and gamma irradiation were explored as proof-of-concept strategies for advanced microtissue preservation, these represented early feasibility assessments only. Formal process validation adhering to ISO and ICH guidelines is required before classifying these as definitive manufacturing or terminal sterilization processes.

A further analytical limitation of the current characterization involves the reporting of GAG content strictly as an absolute value per spheroid, rather than normalizing it to total construct DNA (i.e., µg GAG/µg DNA). While quantifying the absolute GAG content per microtissue is the most clinically relevant metric for defining the final therapeutic payload and structural drug dose prior to implantation, normalizing these biochemical outputs to total DNA would provide a more precise mapping of individual cellular metabolic rates. Future lot-release validations and process characterizations will incorporate specific DNA quantification assays (e.g., utilizing PicoGreen or Hoechst fluorometric protocols) to definitively confirm that the massive ECM accumulation observed under hypoxic 3D conditions is driven by true chondrogenic hyper-synthesis at the single-cell level, rather than merely reflecting cellular proliferation within the construct.

Scientifically, while this study comprehensively defines the in vitro formulation parameters, validates in vitro phenotypic resilience, and demonstrates superior biochemical matrix production compared to baselines, the absence of mechanical testing, ex vivo cartilage defect models, and in vivo efficacy data represents a critical limitation. Without rigorous biomechanical evaluations (such as confined compression testing) or true tissue-integration assays within native host cartilage, claims regarding the clinical readiness or functional superiority of this formulation cannot be substantiated at this stage. While existing literature strongly suggests that 3D matrix implantation yields superior cartilage repair compared to 2D-expanded cell injections, these properties must be explicitly proven for this specific engineered microtissue [75,80]. Therefore, mandatory future studies must evaluate the biomechanical competence, host tissue integration, and long-term efficacy of these spheroids in clinically relevant ex vivo explants and in vivo animal models before clinical translation can be considered [41].

Ultimately, the definitive proof-of-concept for the FE002-Cart engineered microtissue will require well-designed, rigorously controlled, head-to-head human clinical trials directly comparing it against standard interventions and 2D-expanded cell suspensions [22]. These trials must incorporate comprehensive assessments of patient outcomes, utilizing validated metrics to quantify improvements in pain relief and functional recovery, alongside advanced imaging techniques (e.g., 3D-MOCART) to definitively assess long-term structural regeneration [84]. Crucially, to justify the higher manufacturing costs inherent to 3D biomanufacturing, these clinical studies must be paired with a detailed economic analysis. As demonstrated by recent regulatory appraisals, these health technology assessments must rigorously evaluate cost-effectiveness per Quality-Adjusted Life Year (QALY), carefully weighing the therapy’s impact on both direct healthcare expenditures (such as hospitalization, surgical procedures, and rehabilitation) and indirect societal costs (including lost productivity and disability) across different payer systems [4,8,23,91]. By addressing these manufacturing milestones and outlining strict clinical requirements, this research lays a rigorous scientific foundation for the clinical translation of allogeneic FE002-Cart chondroprogenitor spheroids.

## 5. Conclusions

This study provides a comprehensive, optimized framework for manufacturing scaffold-free, allogeneic FE002-Cart chondroprogenitor spheroids targeted for large knee cartilage defects. By precisely defining critical microenvironmental thresholds, specifically physiological hypoxia and restricted glucocorticoid exposure, and rigorously qualifying GMP raw materials, we engineered 3D microtissues with appropriate matrix homogeneity and comparatively superior biosynthetic efficiency compared to traditional matrix-assisted platforms. Crucially, utilizing this highly characterized allogeneic cell source significantly reduces the profound phenotypic unpredictability and batch-to-batch variability inherent in primary autologous chondrocyte therapies, satisfying an absolute regulatory prerequisite for scalable clinical translation.

Beyond establishing biological superiority, this 3D formulation addresses specific in vitro translational and supply-chain challenges. The dense microtissues exhibited robust structural resilience in highly catabolic, simulated osteoarthritic environments while retaining the essential capacity for host tissue integration. Furthermore, we established a highly practical off-the-shelf logistical model, demonstrating that these formulations can be maintained at ambient temperature for up to seven days without compromising their biological potency. Ultimately, by overcoming fundamental biological dependencies and rigid cold-chain constraints, this research lays a rigorous scientific and operational foundation for a standardized, promising engineered microtissue formulation in knee articular cartilage repair.

## Figures and Tables

**Table 1:** Demographic data for the donors of adult articular chondrocytes in the DAL biobank.

Donor ID	Age (Years)	Sex (M/F)	Biopsy Harvest Site (R/L Knee)
A	22	Male	Medial trochlea groove (R)
B	34	Male	Medial trochlea groove (L)
C	28	Female	Intercondylar notch (R)
D	29	Male	Medial trochlea groove (L)
E	20	Female	Medial trochlea groove (L)
F	30	Female	Medial trochlea groove (L)
G	34	Male	Intercondylar notch (R)

**Table 2:** Forward (F) and reverse (R) primer sequences, alongside their respective accession numbers, employed for the quantitative real-time PCR (RT-qPCR) amplification of key chondrogenic markers.

Target Gene	Primer Sequence (5′ → 3′)	Accession No.
*B2M*	F: 5′ CCA CTG AAA AAG ATG AGT ATG CCT 3′ R: 5′ CCA ATC CAA ATG CGG CAT CTT CA 3′	NM004048
*ACAN*	F: 5′ GGT ACC AGT GCA CAG AGG GGT T 3′ R: 5′ TGC AGG TGA TCT GAG GCT CCT 3′	NM 001135
*COL1A1*	F: 5′ AGG GCC AAG ACA TC 3′ R: 5′ AGA TCA CGT CAT CGC ACA ACA 3′	NM 000088
*COL2A1*	F: 5′ GGC AAT AGC AGG TTC ACG TAC 3′ R: 5′ GAT AAC AGT CTT GCC CCA CTT 3′	NM 001844
*COL10A1*	F: 5′ CGC TGA ACG ATA CCA AAT GCC C 3′ R: 5′ TGG ACC AGG AGT ACC TTG CTC T 3′	NM 000493

**Table 3:** Qualitative and semi-quantitative assessment ^1^ of FE002-Cart spheroids cultured under varying hypoxic conditions (2%, 5%, and 10% O_2_). The summarized criteria encompass macroscopic growth, glycosaminoglycan (GAG) accumulation, chondrogenic gene induction, and histological architecture to justify the selection of 2% O_2_ as a critical process parameter. Scoring reflects consistent observations confirmed across *n* = 3 independent biological replicates (manufacturing batches).

Parameters	Criteria for Evaluation	2% O_2_	5% O_2_	10% O_2_	Normoxia
Macroscopic appearance	Increased size over culture time	++	++	+	−
GAGs content (DMMB quantification)	GAGs/spheroids > 5 µg	++	+	−	−
Chondrogenic gene induction	Induction of *COL2* and *ACAN*	++	++	+	−
Histology	Peripheral cell layer: Fibroblastic	++	++	++	+
	Core cells in lacunae: Rounded morphology	++	++	−	−
	AB staining	++	++	+	+
	ACAN immunostaining	++	++	++	+
	COL2 immunostaining	++	+	±	±
	COL10 immunostaining	+/−	+	+	+

^1^ To establish rigorous acceptance criteria, the semi-quantitative scoring symbols represent the following defined numerical thresholds derived from the baseline characterizations: (++) Optimal: Mean Feret diameter > 1.10 mm; mean GAG content > 15 µg/spheroid; gene transcript induction > 200-fold relative to baseline; and strictly homogeneous, dense histological staining. (+) Sufficient: Mean Feret diameter between 0.90 and 1.10 mm; mean GAG content between 5 and 15 µg/spheroid; gene induction between 50- and 200-fold; and positive but variable histological staining. (±) Inconclusive/Highly Variable: Parameters exhibit high intra-batch variance (SD > 20% of the mean) or borderline structural consolidation. (−) Insufficient: Mean Feret diameter < 0.90 mm; mean GAG content < 5 µg/spheroid; severely blunted transcriptomic induction (< 50-fold); and absent or negligible histological staining.

**Table 4:** Quantitative proteomic characterization of the cellular active substance under distinct oxygen culture conditions. The table displays the normalized relative protein concentrations (ng/mg) within the soluble fractions of FE002-Cart cell lysates cultured in normoxia or defined hypoxia (2%, 5%, and 10% O_2_). Only proteins with a detected concentration of ≥ 0.5 ng/mg are included. Quantitative data are presented as the mean ± standard deviation of *n* = 3 independent manufacturing batches per atmospheric condition.

Abbreviated Protein Name	Full Protein Name	Normalized Relative Protein Concentration in the Cell Lysate Soluble Fraction (ng/mg)			
		2% O_2_	5% O_2_	10% O_2_	Normoxia
HMGB1	High mobility group protein B1	40.22 ± 3.81	33.62 ± 2.95	29.50 ± 5.27	23.55 ± 2.60
TIMP-2	Tissue inhibitor of metalloproteinase 2	8.35 ± 1.48	9.77 ± 2.64	12.63 ± 2.18	12.40 ± 3.95
sEGFR	Soluble epidermal growth factor receptor	6.92 ± 0.98	4.40 ± 1.62	6.35 ± 2.04	5.76 ± 1.39
TIMP-1	Tissue inhibitor of metalloproteinase 1	5.34 ± 1.48	3.63 ± 1.64	3.05 ± 0.73	4.45 ± 1.49
MMP-2	Matrix metalloproteinase 2	2.77 ± 0.75	3.79 ± 0.45	8.12 ± 1.63	3.97 ± 1.97
Follistatin	Follistatin	5.94 ± 1.94	2.13 ± 2.59	0.87 ± 0.29	2.74 ± 0.85
FGF-2	Fibroblast growth factor 2	3.00 ± 0.73	2.63 ± 0.91	1.95 ± 0.85	1.98 ± 0.80
Sgp130	Soluble gp130	2.69 ± 0.84	1.58 ± 0.99	1.48 ± 1.08	1.58 ± 1.08
M-CSF	Macrophage colony-stimulation factor	1.92 ± 0.47	2.08 ± 0.29	2.01 ± 0.19	1.39 ± 0.78
sTNFRI	Soluble tumor necrosis factor receptor I	1.15 ± 0.15	0.93 ± 0.25	1.12 ± 0.61	0.71 ± 0.44
HGF	Hepatocyte growth factor	0.98 ± 0. 11	0.49 ± 0.09	1.01 ± 0.17	0.42 ± 0.08

**Table 5:** Morphological, transcriptional, and biochemical benchmarking of 3D microtissues engineered from the allogeneic FE002-Cart chondroprogenitor cell type versus primary adult human chondrocytes derived from seven independent donors (Donors A–G). All cellular cohorts were subjected to identically optimized manufacturing parameters, which included 2D hypoxic pre-conditioning followed by scaffold-free aggregation at 2% O_2_ in the presence of 10 nM dexamethasone. Quantitative data are presented as the mean ± standard deviation of *n* = 3 independent manufacturing batches per cell source. Statistical comparisons were performed exclusively on the averaged batch-level data to eliminate technical pseudoreplication.

Parameter	FE002-Cart	Donor A	Donor B	Donor C	Donor D	Donor E	Donor F	Donor G
Area (mm^2^)	1.21 ± 0.08	0.76 ± 0.12	1.45 ± 0.18	0.59 ± 0.10	0.99 ± 0.07	1.42 ± 0.26	0.88 ± 0.11	1.09 ± 0.14
Circularity	0.60 ± 0.06	0.42 ± 0.11	0.49 ± 0.10	0.50 ± 0.03	0.61 ± 0.06	0.49 ± 0.04	0.38 ± 0.06	0.52 ± 0.09
Feret Diameter (mm)	1.26 ± 0.08	0.94 ± 0.07	1.62 ± 0.12	0.99 ± 0.03	1.06 ± 0.02	1.56 ± 0.06	1.16 ± 0.12	1.28 ± 0.04
*COL1A1* Fold Induction	0.97 ± 0.2	1.5 ± 0.5	5.8 ± 1.2	14.0 ± 2.9	32.9 ± 6.1	9.4 ± 1.8	6.2 ± 0.7	2.0 ± 0.2
*COL10A1* Fold Induction	1.4 ± 0.3	29 ± 4	258 ± 48	1137 ± 148	723 ± 82	490 ± 16	70 ± 3	211 ± 9
Mean GAGs/Spheroid (µg)	22 ± 4	18 ± 3	29 ± 5	12 ± 2	13 ± 1	46 ± 5	9 ± 2	15 ± 3

**Table 6:** Systematic assessment ^1^ of spheroid quality attributes during the transition to a GMP-compliant manufacturing process. The table evaluates the biological impact, encompassing macroscopic development, glycosaminoglycan (GAG) content, chondrogenic gene expression, and histological architecture of the spheroids after substituting (one component substitution each time) standard research-grade reagents with various pharmaceutical-grade formulations of insulin, vitamin C, and dexamethasone. ✓, appropriate; ±, inconclusive, highly variable. Scoring reflects consistent observations confirmed across *n* = 3 independent manufacturing batches.

Parameters	Criteria for Evaluation ^1^	ITS		Insulin					Vitamin C	Dexamethasone		
		X	G	Novorapid	FIASP	Apidra	Humalog	Lyumjev	Streuli	Mephameson	Fortecortin	Tocris
Macroscopic	Size	✓	✓	✓	✓	✓	✓	±	✓	±	±	✓
GAG Content	GAGs/Spheroids	✓	✓	✓	✓	✓	✓	±	✓	±	±	✓
Chondrogenic Gene Induction	*ACAN*	✓	✓	✓	±	±	✓	✓	✓	✓	±	✓
	*COL2*	✓	✓	✓	✓	✓	✓	✓	✓	±	±	✓
Histology	Peripheral Cell Layer: Fibroblastic	✓	✓	✓	✓	✓	✓	✓	✓	✓	✓	✓
	Core Cells in Lacunae: Rounded Morphology	✓	✓	✓	✓	✓	✓	✓	✓	±	±	✓
	AB staining	✓	✓	✓	✓	✓	✓	✓	✓	✓	✓	✓
	ACAN staining	✓	✓	✓	✓	✓	✓	✓	✓	✓	✓	✓

^1^ To eliminate subjectivity and determine the non-inferiority of the GMP-compatible components, the scoring symbols denote strict numerical acceptance criteria evaluated against the baseline research-grade control: (✓ Appropriate): All quantitative parameters (macroscopic size, GAG content, and *ACAN*/*COL2* fold-induction) strictly fall within ± 15% of the research-grade baseline means. For qualitative histology, this denotes a blinded operator confirmation of spatial matrix distribution equivalent to the baseline. (± Inconclusive/Highly Variable): Quantitative parameters deviate strictly by > 15% from the research-grade baseline (e.g., significant macroscopic size reductions) or exhibit a standard deviation > 20% across batches.

**Table 7:** Evaluation of short-term liquid storage parameters (24 h, 22 °C) on the logistical viability and functional integrity of engineered 3D FE002-Cart spheroids. Following manufacturing maturation, microtissues were suspended in standard clinical transport solutions (0.9% NaCl, Ringer’s solution, PBS, DPBS, and 5% glucose), with select formulations supplemented with 2% human serum albumin (HSA) and compared against a minimal basal medium (DMEM). The preservation of critical quality attributes was comprehensively assessed via cellular metabolic viability (MTT reduction and quantification), ECM retention (total GAG content), and the maintenance of autonomous inter-spheroid fusion capacity. ✓, appropriate. Results marked “oos” (out of specification) indicate failure to support the metabolic demands of the dense 3D constructs. All storage parameters were systematically evaluated across *n* = 3 independent manufacturing batches to confirm the reproducibility of the out-of-specification (oos) events.

Parameter	NaCl 0.9%	NaCl 0.9% + HSA 2%	Ringer + HSA 2%	Glucose 5% + HSA 2%	PBS	DPBS	DMEM
MTT Viability	oos	oos	oos	oos	oos	oos	✓
GAG Content (µg/spheroid)	oos	oos	oos	oos	oos	oos	✓
Fusion Capacity	oos	oos	oos	oos	oos	oos	✓

**Table 8:** Temporal and temperature-dependent stability profiling of formulated 3D FE002-Cart spheroids for short-term logistical viability determination. Following manufacturing maturation, engineered microtissues were maintained in DMEM minimal basal medium at either standard refrigerated conditions (4 °C) or ambient temperature (22 °C) and evaluated at 2, 4, and 7 days post-harvest. The preservation of critical quality attributes was systematically assessed by determining cellular metabolic activity (MTT reduction and quantification), ECM retention (total GAG content), and autonomous inter-spheroid fusion capacity. ✓, appropriate. Results marked “oos” (out of specification) indicate failure to support the metabolic demands of the dense 3D constructs. Stability outcomes reflect observations from n=3 independent manufacturing batches assessed at each designated timepoint and temperature configuration.

Storage Duration	Storage Conditions	Parameters	Outcomes
2 days	4 °C	MTT	oos
		GAG Content	oos
		Fusion	oos
	22 °C	MTT	✓
		GAG Content	✓
		Fusion	✓
4 days	4 °C	MTT	oos
		GAG Content	oos
		Fusion	oos
	22 °C	MTT	✓
		GAG Content	✓
		Fusion	✓
7 days	4 °C	MTT	oos
		GAG Content	oos
		Fusion	oos
	22 °C	MTT	✓
		GAG Content	✓
		Fusion	✓

## Data Availability

The data presented in this study are available in the article files.

## Abbreviations

AB	Alcian blue
ACAN	aggrecan
ACI	autologous chondrocyte implantation
ANOVA	analysis of variance
ATMP	advanced therapy medicinal product
BCA	bicinchoninic acid
cDNA	complementary DNA
CHUV	Centre hospitalier universitaire vaudois
CPP	critical process parameters
Ct	cycle threshold
DD	degenerative disease
DMEM	Dulbecco’s modified Eagle medium
DMMB	dimethylmethylene blue assay
ECM	extracellular matrix
FBS	fetal bovine serum
FGF	fibroblast growth factor
GAG	glycosaminoglycan
GMP	good manufacturing practices
h	hour
H&E	Hematoxylin and Eosin
HGF	hepatocyte growth factor
HIF-1	hypoxia-inducible factor
HMGB1	high mobility group protein B1
HRP	horseradish peroxidase
HSA	human serum albumin
HSD	honestly significant difference
ICH	International Council for Harmonisation
IHC	immunohistochemistry
IL	interleukin
ITS	insulin, transferrin, selenous acid
KOOS	Knee injury and Osteoarthritis Outcome Score
MACI	matrix induced autologous chondrocyte implantation
M-CSF	macrophage colony-stimulation factor
MFX	microfracture
MMPs	matrix metalloproteinases
MSC	mesenchymal stem cells
MTT	3-(4,5-dimethylthiazol-2-yl)-2,5-diphenyltetrazolium bromide
NaCl	sodium chloride
N-CAM	undifferentiated nasal chondrocytes
NEAA	non-essential amino acids
N-TEC	mature tissue-engineered cartilage
OA	osteoarthritis
oos	out of specification
PBS	phosphate-buffered saline
Ph. Eur.	European Pharmacopoeia
QC	quality control
QALY	quality-adjusted life year
RT-qPCR	quantitative real-time polymerase chain reaction
SDS-PAGE	sodium dodecyl sulfate-polyacrylamide gel electrophoresis
sEGFR	soluble epidermal growth factor receptor
Sgp130	soluble gp130
sTNFRI	soluble tumor necrosis factor receptor I
TGF	transforming growth factor
TIMP	tissue inhibitor of metalloproteinase
TNF	tumor necrosis factor
UK	United Kingdom
USA	United States of America
VitCp	vitamin C 2-phosphate sesquimagnesium salt hydrate
